# West Nile virus unmasked: from gene variability to future challenges

**DOI:** 10.3389/fcimb.2025.1690827

**Published:** 2025-11-05

**Authors:** Samuel Prieto-Vega, Alfredo Berzal-Herranz, Juan José Garrido, Armando Arias, Ana Grande-Pérez, Ana María Fernández-Escamilla, María Montoya

**Affiliations:** ^1^ Virus Evolution Group, Departamento de Biología Celular, Genética y Fisiología, Facultad de Ciencias, Universidad de Málaga, Málaga, Spain; ^2^ Instituto de Parasitología y Biomedicina “López-Neyra” (IPBLN), CSIC, PTS, Granada, Spain; ^3^ Immunogenomics and Molecular Pathogenesis Group, UIC Zoonoses and Emergent Diseases ENZOEM, Department of Genetics, University of Córdoba, Córdoba, Spain; ^4^ Maimónides Biomedical Research Institute of Córdoba (IMIBIC), GA-14 Research Group, Córdoba, Spain; ^5^ Instituto de Biomedicina de UCLM (IB-UCLM), Unidad de Biomedicina UCLM-CSIC, Escuela Técnica Superior de Ingenieros Agrónomos y de Montes y Biotecnología (ETSIAMB), Universidad de Castilla-La Mancha (UCLM), Albacete, Spain; ^6^ Instituto de Investigación, Desarrollo e Innovación en Biotecnología Sanitaria de Elche (IDiBE), Universitas Miguel Hernández, Elche, Alicante, Spain; ^7^ Viral Immunology Lab, Biomedicine Department, Margarita Salas Center for Biological Research (CIB-CSIC), Bic Unit, Madrid, Spain; ^8^ Unit for the Development of Biological, Immunological and Chemical Drugs, Centro de Investigaciones Biológicas Margarita Salas (CSIC), Madrid, Spain

**Keywords:** West Nile virus, Orthoflavivirus, genetic variability, therapeutics, host-virus interaction, West Nile fever

## Abstract

West Nile virus (WNV) is a mosquito-borne orthoflavivirus with a complex transmission cycle involving avian reservoirs and mosquito vectors. Although no precise global infection figure exists, conservative estimates based on seroprevalence data suggest between 4 and 16 million infections annually. With an approximate mortality rate of 6–7% among reported cases, WNV poses a significant public health concern across continents. This review provides a comprehensive overview of WNV molecular biology, including genome organization, protein maturation, replication mechanisms, the functional roles of untranslated regions (UTRs) and post-translational modifications in viral adaptation. Particular attention is given to intrahost genetic variability and the quasispecies nature of WNV as key drivers of immune evasion and viral evolution. The ecological and epidemiological dynamics of WNV are also discussed in the context of climate change and its impact on vector distribution and global viral spread. Additionally, the review details clinical manifestations, pathogenesis, diagnostic tools, and current therapeutic strategies. Emerging approaches for prevention and control are explored, including entomological surveillance, vaccine development, and novel antiviral candidates such as targeted peptides, antibodies and lethal mutagenesis. Given the pressing challenges associated with WNV, this review underscores the importance of integrated One Health surveillance systems and accelerated vaccine development to mitigate future outbreaks, highlighting the intersection of virology, immunology, ecology, and global health.

## Graphical Abstract

Overview of West Nile virus (WNV) biology, epidemiology, and control. The figure summarizes viral genome and proteins, replication cycle, intra-host variability, and epidemiological transmission. It also highlights clinical disease, prevention and control strategies, and current as well as experimental anti-WNV approaches.

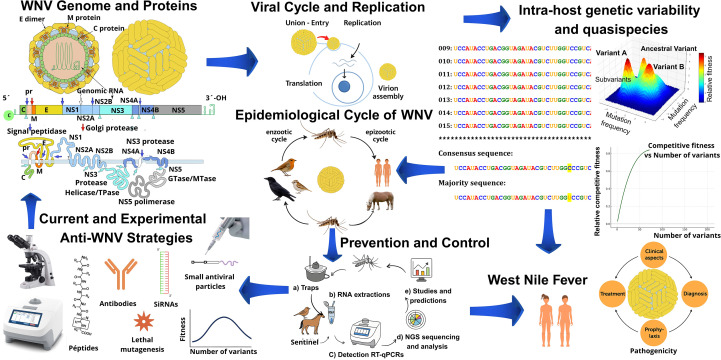

## 1 Introduction

WNV *(Orthoflavivirus nilense)* is a member of the family *Flaviviridae* and genus *Orthoflavivirus* (Postler et al., 2023), and was first identified in Uganda in 1937 (Smithburn et al., 1940; Campbell et al., 2002). Since its discovery, WNV has demonstrated remarkable ecological adaptability and geographic expansion, with significant outbreaks reported across multiple continents since the 1950s (Hayes, 2001; Chancey et al., 2015; Sule et al., 2018). A major milestone in its global emergence occurred with its introduction to North America in the late 1990s. During the 1999 outbreak in New York City, 62 clinical cases and 7 fatalities due to WNV-induced meningoencephalitis were reported. By 2002, approximately 4,000 human cases and 250 deaths had been recorded, along with widespread avian mortality—particularly among crows (Powell, 2003)—and WNV had spread across the continental United States (US). The virus subsequently disseminated globally, establishing itself as a pathogen of increasing concern in Europe, Asia, and South America (Nash et al., 2001). Although definitive evidence remains elusive, the rapid transcontinental spread of WNV is most plausibly attributed to migratory bird pathways and animal trade, with additional contributions from the movement of humans and goods from the US. In North America, WNV has caused substantial mortality among equines, birds, and humans, with 2,958 confirmed human deaths between 1999 and 2013, and 182 deaths reported in 2023 alone, according to the CDC’s “Final Annual Maps & Data for 1999–2023” (CDC, 2025). These data underscore the virus’s significant impact on global public health.

WNV is primarily transmitted by ornithophilic mosquitoes of the genus *Culex*, with avian species serving as the principal natural reservoirs (Hubálek and Halouzka, 1999; Turell et al., 2005; Pfeffer and Dobler, 2010). Humans and equines are considered incidental, dead-end hosts (Schwarz and Long, 2023). In both humans and equids, WNV infection can lead to West Nile encephalitis (Ulbert, 2011; Garcia et al., 2018; Byas and Ebel, 2020; Habarugira et al., 2020; Schwarz and Long, 2023). While most human infections are asymptomatic or present with mild febrile illness, a minority, particularly the elderly or immunocompromised, may progress to severe neuroinvasive disease, including encephalitis and meningitis (David et al., 2007; Kumar et al., 2016; Kumar et al., 2018; Tomar et al., 2022).

At the genetic level, WNV populations are considered quasispecies, a dynamic ensemble of genetically related variants acting as a unit of natural selection (Jerzak et al., 2005; Van Slyke et al., 2012). Although viral diversification is generally constrained within mosquito vectors (Brackney et al., 2015), certain avian species, particularly Passeriformes, such as corvids are highly susceptible to infection and can serve as amplification hosts. These distinctive biological features have positioned WNV as a valuable model for studying viral evolution, transmission, and pathogenesis (Lee et al., 2013; Garcia et al., 2018; Vouillon et al., 2024).

Unlike recent reviews of WNV that have focused primarily on epidemiological trends, clinical presentations, and vaccine development (Suthar et al., 2013b; Chancey et al., 2015; Patel et al., 2015; Habarugira et al., 2020; Ronca et al., 2021; Gould et al., 2025; Kocabiyik et al., 2025), this manuscript offers an updated synthesis of recent advances in WNV quasispecies dynamics, host-immune interactions, viral evasion mechanisms, and molecular pathogenesis. We introduce and discuss population dynamics frameworks, specifically error catastrophe and lethal defection, as emerging conceptual tools that offer novel insights for WNV surveillance and control. Thus, this work expands the current literature by integrating state-of-the-art theoretical models and highlighting unique aspects of viral population behavior not addressed in existing reviews. (Graphical abstract).

## 2 Viral genome and proteins

**Figure 1:**
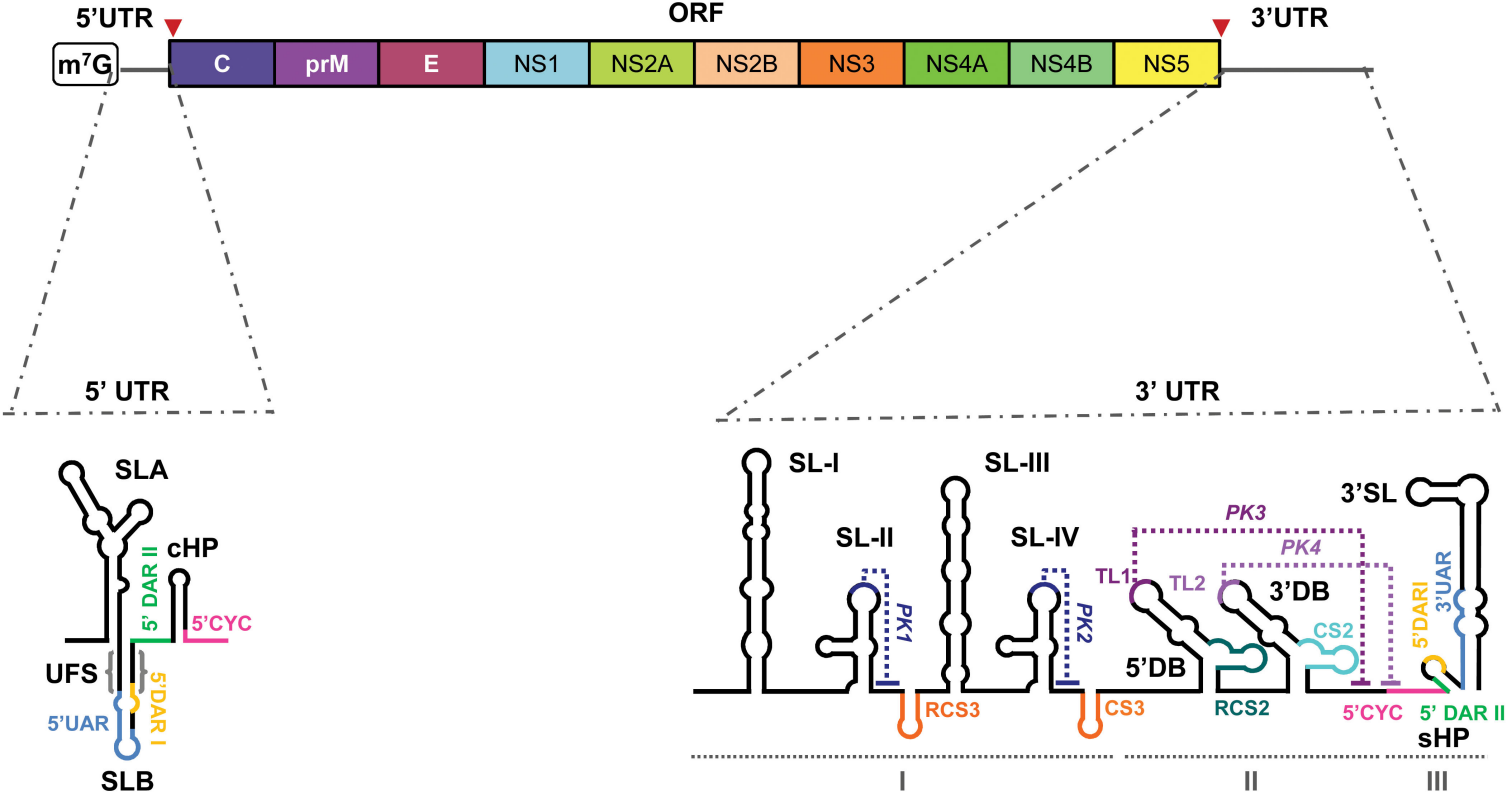
WNV genome organization. Schematic representation of the viral genome structure, featuring the 5' and 3' untranslated regions (UTR), and the single open reading frame (ORF) of the WNV genome. Different colors delimit the coding sequence of each viral protein, including the structural (C, prM/M, E) and nonstructural proteins (NS1–NS5). Below, two detailed schematic representations of the secondary structure of the two UTRs. They depict the main RNA structural elements: stem-loops (SLA, SLB, cHP, SL-I, SL-II, SL-III, SL-IV, 3'SL, RCS3, CS3, RCS2, CS2 and sHP); dumbbell elements (5ʹDB and 3ʹDB) and pseudoknots (PKI–PKIV) indicated with dotted lines. Cyclization sequences (UAR, DARI, DARII and CYC) are indicated with colored lines. Other functional sequence motifs (TL1 and TL2) are also indicated. The structural domains into which the 3ʹ UTR is divided are indicated by I–III. Red arrowheads indicate the translation start and stop codons.

The WNV genome consists of a single-stranded positive-sense RNA molecule approximately 11,000 nucleotides (nt) in length. It contains a single open reading frame (ORF) flanked by highly structured 5’ and 3’ untranslated regions (UTRs), which play crucial roles in the viral replication cycle (**Figure 1**).

### 2.1 Genome of WNV

WNV ORF codes for a single polyprotein that undergoes co- and post-translational processing yielding 10 mature viral proteins (Nowak et al., 1989). They can be classified in NS (NS1, NS2A, NS2B, NS3, NS4A, NS4B, and NS5) and S proteins [capsid (C), premembrane/membrane (prM/M), and envelope (E)] (Chambers et al., 1990). This genomic organization is conserved among the members of the genus *Orthoflavivirus.*

Besides storing genetic information and being the replication template, it also acts as the only viral messenger RNA (mRNA) and behaves as a succession of cis-acting ncRNA-like molecules that perform functions themselves (Brinton et al., 1986; Brinton and Dispoto, 1988; Brinton, 2013; Fernández-Sanlés et al., 2017; Ng et al., 2017). The RNA bears a type 1 cap at its 5’ end (m7GpppAmp) (Ray et al., 2006; Zhou et al., 2007; Saeedi and Geiss, 2013) but it lacks a polyA tail at the 3’ end (Wengler and Wengler, 1981; Brinton et al., 1986), instead it ends with a conserved CU-OH dinucleotide. Although the existence of a cap-independent translation initiation mechanism has been suggested (Berzal-Herranz et al., 2022), an internal ribosome entry site (IRES) has not been identified in the genome, which distinguishes orthoflaviviruses from members of the other genera of the family *Flaviviridae*. In addition to the protein-coding information viral RNA genomes store information in structurally conserved RNA elements scattered throughout the genome, which perform their own functions (Huston et al., 2024).

### 2.2 Untranslated regions

WNV UTRs are complex folded regions consisting of a succession of RNA structural elements. They are structured RNA regions comprising consecutive RNA elements that form long-range RNA-RNA interactions, providing the genomic RNA scaffold and regulating viral processes via the RNA-protein interactome.

5’ UTR: The ~100-nt 5’ UTR contains two conserved stem-loops (Markoff, 2003) (**Figure 1**):

- SLA (~70 nt) adopts a Y-shaped fold with a side stem-loop (SSL) and mediates NS5 polymerase recruitment and 5’ capping during replication (Filomatori et al., 2006; Zhang et al., 2012; Fernández-Sanlés et al., 2017).
- SLB, located downstream, contains the AUG start codon and flanking 5’UAR and 5’DAR sequences required for genome cyclization, a critical step in the viral cycle (Fernández-Sanlés et al., 2017).

3’ UTR: The ~700-nt 3’ UTR ends in a conserved CU-OH and features tandemly duplicated RNA elements linked to host adaptation (de Borba et al., 2019) (**Figure 1**). It is organized in three domains:

- Domain I: Contains duplicated stem-loops (SLI-SLII/SLIII-SLIV) and CS3 elements forming pseudoknots (PK1/PK2) that stall XRN1 and generate sfRNAs, influencing viral infectivity, host adaptation, and immune evasion (Pijlman et al., 2008; Roby et al., 2014; Ramos-Lorente et al., 2021).
- Domain II: Comprises two dumbbell elements (5’DB, 3’DB) essential for replication, translation regulation, 40S ribosomal subunit recruitment, and sfRNA production (Funk et al., 2010; Manzano et al., 2011; Clarke et al., 2015; de Borba et al., 2019; Berzal-Herranz et al., 2022; Ramos-Lorente et al., 2024).
- Domain III: Includes a short hairpin (sHP) and the 3’SL stem-loop (~80–90 nt), essential for replication via NS5 recruitment at the 5’-CACAG-3’ motif, translation regulation, and genome cyclization (Gritsun et al., 1997; Khromykh et al., 2003; Elghonemy et al., 2005; Filomatori et al., 2011; Ochsenreiter et al., 2019; Ramos-Lorente et al., 2024).

### 2.3 WNV proteins

**Figure 2:**
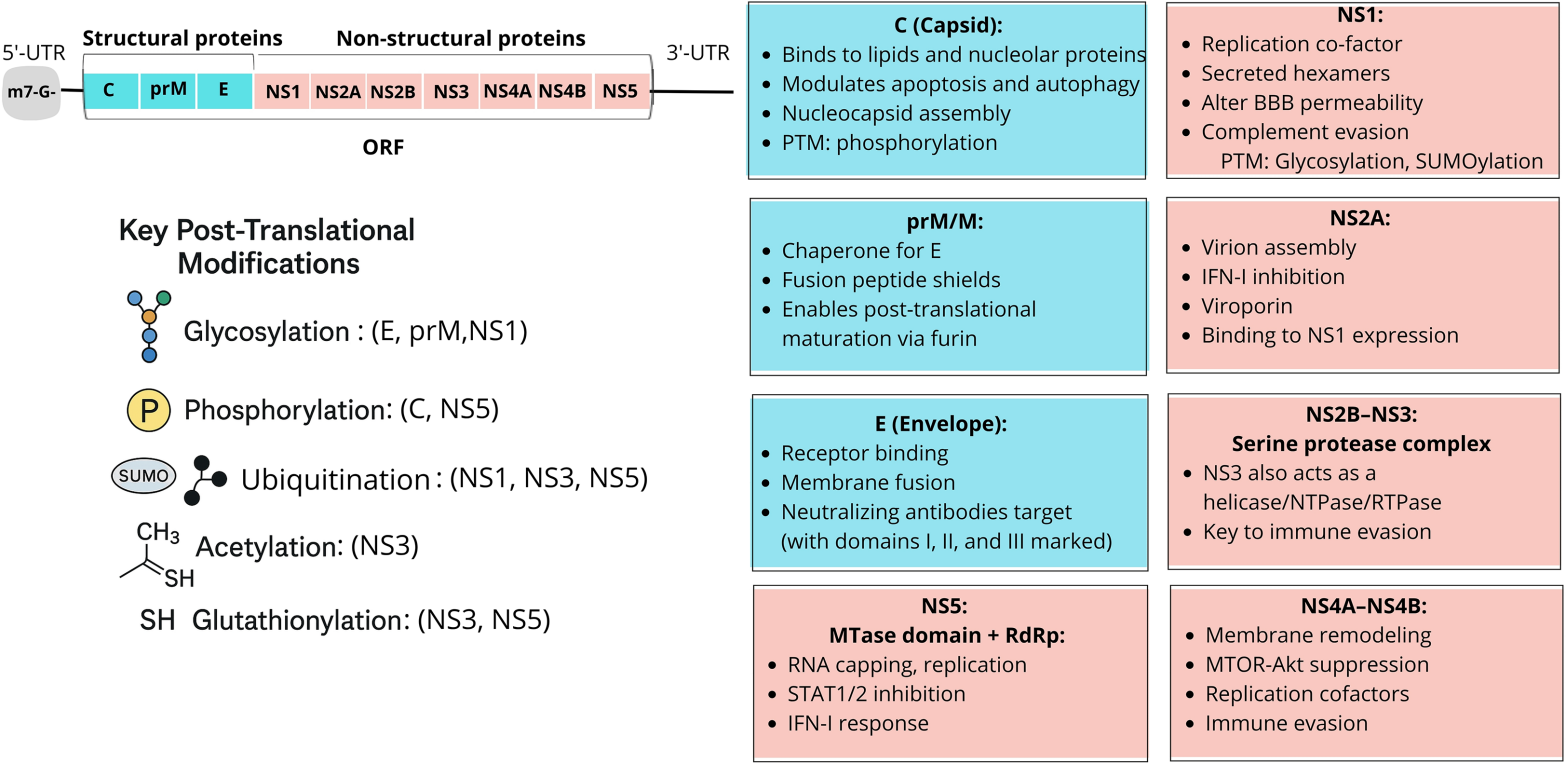
Schematic representation of WNV structural (S) and non-structural (NS) proteins and their main functional roles. Top right: the WNV genome encodes a single polyprotein that is cleaved into three S proteins (C, prM/M, E) and seven NS proteins (NS1–NS5), each with distinct functions. Below: key posttranslational modifications (PTMs). Major PTMs regulate the structure and function of WNV proteins. Glycosylation affects the envelope (E), premembrane (prM), and NS1, influencing virion assembly, secretion, and immune evasion. Phosphorylation modulates capsid (C) protein function in nucleocapsid formation and NS5 activity in RNA replication and nuclear translocation. SUMOylation and ubiquitination target NS1, NS3, and NS5, altering stability, localization, and interactions with host immune pathways. Acetylation and glutathionylation (GSH) affect NS3 and NS5, regulating enzymatic activities such as helicase and polymerase function, thereby contributing to replication fitness. These PTMs are critical for WNV pathogenesis and may represent potential antiviral targets. Boxes: principal protein functions. Capsid (C) mediates nucleocapsid assembly and modulates apoptosis; prM/M acts as a chaperone for E and ensures maturation; E drives receptor binding and membrane fusion. Among non-structural proteins, NS1 is a replication cofactor that disrupts the blood–brain barrier; NS2A, NS4A, and NS4B contribute to replication and immune evasion; NS2B–NS3 forms the protease–helicase complex; and NS5 encodes the RNA-dependent RNA polymerase and methyltransferase essential for replication and innate immune antagonism.

As described above, the WNV genome encodes a single polyprotein that is cleaved into ten proteins exhibiting significant homology with those of other orthoflavivirus species, including important human pathogens such as Dengue virus (DENV *Orthoflavivirus denguei*), Zika (ZIKV *Orthoflavivirus zikaense*), yellow fever (YFV *Orthoflavivirus flavi*), and Japanese encephalitis viruses (JEV *Orthoflavivirus japonicum*). This homology is evident in both structural (S) and nonstructural (NS) proteins, with certain regions highly conserved and others more variable (Zaccaria et al., 2021; Maloney et al., 2023). A schematic summary of WNV proteins depicted in **Figure 2**.

#### 2.3.1 WNV structural proteins

##### 2.3.1.1 Capsid protein

The capsid (C) protein primarily functions in binding and packaging the viral RNA genome into the nucleocapsid, a process essential for virion assembly and budding through association with host membranes (Zhang et al., 2003; Brinton, 2013; Acharya and Bai, 2016; Karim and Bai, 2023). It may also facilitate genome delivery during viral entry following membrane hemifusion (Mukhopadhyay et al., 2005). Beyond structural roles, the WNV C protein contributes to viral replication and host interaction by associating with lipid droplets and very low-density lipoproteins (VLDL) (Zhang et al., 2003; Martins et al., 2019), binding host factors such as DDX56 required for infectious particle assembly (Xu et al., 2011), and translocating to the nucleus to influence host functions (Mukhopadhyay et al., 2003; Dokland et al., 2004).

C protein further modulates cell death pathways, inducing apoptosis via HDM2 sequestration in a p53-dependent manner (Yang et al., 2008), while early in infection, it blocks apoptosis through PI3K/Akt activation to support replication (Zhang et al., 2003; Urbanowski and Hobman, 2013). It also disrupts autophagy by promoting AMPK ubiquitination and degradation, a mechanism linked to neuropathology and efficient virion assembly (Mukhopadhyay et al., 2003; Kobayashi et al., 2020). Evidence from related flaviviruses (e.g., ZIKV) suggests additional roles in immune evasion, including inhibition of the integrated stress response (ISR) and suppression of RNA silencing via interference with Dicer activity, thereby enhancing replication and avoiding immune detection (Dokland, 2000; Dokland et al., 2004; Samuel and Diamond, 2006; Bogachek et al., 2007; Bhuvanakantham et al., 2009; Blázquez et al., 2021; Alghamdi et al., 2025).

Premembrane (prM/M) Protein: The pre-membrane (prM) protein of WNV serves as a chaperone, ensuring the correct folding of the envelope (E) protein during the assembly of new viral particles, while also preventing premature fusion by masking the E protein’s fusion peptide (Setoh et al., 2012; Fiacre et al., 2020). In immature virions, prM forms heterodimers with E, creating spike-like structures on the viral surface. The protective role of prM is crucial during virion synthesis, as it shields the E protein from premature activation within the acidic environment of the trans-Golgi network, thereby aiding in proper viral assembly and secretion, and may contribute to immune evasion and virulence (Mukhopadhyay et al., 2005; Zhang et al., 2013; Stoddard et al., 2015; Pierson and Diamond, 2020).

The envelope (E) protein is a glycoprotein essential for host cell infection, mediating receptor binding and the fusion of viral and host membranes under acidic endosomal conditions, which enables genome release into the cytoplasm (Nybakken et al., 2006; Kaufmann and Rossmann, 2011; Alsaleh et al., 2016; Zhang et al., 2017; Pierson and Diamond, 2020). Viral attachment occurs in a cell-type-specific manner via host membrane factors, with distinct E protein domains, including the conserved fusion peptide, playing critical roles in entry (Chu and Ng, 2004; Nybakken et al., 2005; Nelson et al., 2008; Martin and Nisole, 2020; Villalaín, 2025). Structurally, the E protein comprises three domains: DI for stability, DII containing the fusion loop, and DIII responsible for receptor binding and serving as a major target of neutralizing antibodies (Nybakken et al., 2006; Valente RP da et al., 2020; Kocabiyik et al., 2025).

E protein is synthesized in the ER, where it forms prM–E heterodimers involved in virion budding. During Golgi transit, low pH induces dissociation of prM and reorganization into E homodimers, while subsequent acidification in endosomes drives E protein rearrangement from dimers to fusogenic trimers (Chu and Ng, 2004). Glycosylation of E protein influences viral attachment and immune evasion (Weiß et al., 2023). As the major antigenic determinant of WNV, epitopes in DIII are primary targets for neutralizing antibodies, while DI epitopes may also be exposed during conformational transitions (Beasley and Barrett, 2002; Rebollo et al., 2018). The conserved fusion peptide in DII mediates membrane fusion across hosts (Volk et al., 2004; Seligman, 2008). Dynamic motion and conformational flexibility of E proteins further underscore their role as central targets for vaccine and therapeutic development (Beasley and Barrett, 2002; Dowd and Pierson, 2018).

#### 2.3.2 WNV non-structural proteins

##### 2.3.2.1 Protein NS1

NS1 is a conserved multifunctional glycoprotein essential for replication, immune evasion, and pathogenesis (Rastogi et al., 2016). It exists in both membrane-associated and secreted forms: intracellularly, NS1 homodimers act as cofactors for RNA replication and colocalize with dsRNA replicative intermediates, while secreted NS1 forms hexameric lipoprotein complexes that associate with host membranes (Youn et al., 2013). Structurally, NS1 is a rod-shaped dimer composed of a 16-stranded β-platform with protruding loops that serve as antibody epitopes, containing conserved N-linked glycosylation sites and cysteines critical for viral viability (Brinton, 2013). NS1 secretion correlates with disease severity and occurs independently of virion release, being detectable in Vero cells 16–24 h post-infection (Macdonald et al., 2005; Chung et al., 2006; Brinton, 2013).

Functionally, NS1 modulates host immunity by binding complement regulators such as factor H, thereby suppressing complement activation and preventing viral clearance (Chung et al., 2006). It also promotes endothelial hyperpermeability of the blood-brain barrier, facilitating neuroinvasion (Beltrami et al., 2025). NS1-specific antibodies provide protection in animal models through Fc-dependent and independent mechanisms (Chung et al., 2006; Nybakken et al., 2006). Moreover, NS1 inhibits RLR-induced IFN-β production by targeting TBK1 and stabilizes caspase-1 via USP8 recruitment, which in turn cleaves cGAS and suppresses DNA sensing pathways (Quicke and Suthar, 2013). Collectively, NS1 supports viral replication, immune evasion, and neuropathogenesis, highlighting its value as a diagnostic and therapeutic target.

##### 2.3.2.2 Alternative protein NS1’

WNV uses a programmed -1 ribosomal frameshift (-1PRF) mechanism during open reading frame (ORF) translation to produce an extended version of NS1, known as NS1’, which enhances neuroinvasiveness and viral RNA abundance (Moomau et al., 2016). Variations in NS1’ synthesis due to dynamic structural rearrangements may account for strain-specific differences in pathogenicity. Formation of the -1 PRF signal, stimulated by a tandem stem-loop evolving into a pseudoknot structure, leads to altered S and NS protein ratios, thereby influencing virion production. Disruption of the −1PRF signal reduces the E/NS5 protein ratio and impairs viral replication (Melian et al., 2014).

Protein NS2A: This is a small hydrophobic, ER-associated protein (~22–25 kDa, ~231 amino acids) that performs multiple roles in the viral replication cycle (Mackenzie et al., 1998; Kümmerer and Rice, 2002). It facilitates RNA replication by binding the 3’ UTR and interacting with replication complex components such as NS3 and NS5 (Liu et al., 2006; Leung et al., 2008). NS2A also coordinates virion assembly by recruiting genomic RNA, structural proteins, and the NS2B/NS3 protease complex to assembly sites, while acting as a viroporin to induce membrane rearrangements (Mackenzie et al., 1998; Leung et al., 2008).

In addition to replication and assembly, NS2A antagonizes host antiviral responses by suppressing type I IFN induction and blocking MDA5-mediated signaling (Liu et al., 2006; Gack and Diamond, 2016). The A30P substitution reduces virulence and cytopathic effect by impairing IFN antagonism and apoptosis regulation (Rossi et al., 2007). Codon 30 is also critical for the pseudoknot structure required for -1 ribosomal frameshifting and NS1’ synthesis, linking NS2A to replication and pathogenesis (Firth and Atkins, 2009; Melian et al., 2014). NS2A interacts with NS4A and other NS proteins to stabilize replication complexes (Leung et al., 2008). Additionally, NS2A may disrupt adherent junctions and radial glial proliferation, contributing to neuropathology as observed in ZIKV (Yoon et al., 2017).

##### 2.3.2.3 Protein NS2B

this is a small hydrophobic membrane-associated protein that functions as an essential cofactor for the NS3 serine protease. The NS2B–NS3 complex cleaves the viral polyprotein at multiple sites, releasing structural and nonstructural proteins required for replication and assembly (Erbel et al., 2006; Chappell et al., 2008; Su et al., 2009). NS2B is necessary for NS3 activation and also recruits and localizes it to the rough ER, where replication complexes form and protease activity is required (Tseng et al., 2021). In the absence of NS2B, NS3 remains cytoplasmic and inactive, underscoring NS2B’s dual role in protease activation and targeting.

Structurally, NS2B contains a conserved central hydrophilic region (~40 amino acids) essential for cofactor function, flanked by hydrophobic regions that anchor the NS2B–NS3 complex to ER membranes, allowing access to the polyprotein substrate (Chappell et al., 2008; Su et al., 2009; Wahaab et al., 2022). Crystal structures show NS2B stabilizes the NS3 protease domain in a catalytically active form (RCSB PDB 5IDK), and disruption of this activity is lethal for replication, making it a prime antiviral target (Chappell et al., 2008; Kang et al., 2013). Evidence from DENV and ZIKV confirms the conserved requirement of NS2B–NS3 interactions across flaviviruses (Phoo et al., 2016).

##### 2.3.2.4 Protein NS3 (serine protease)

This is a multifunctional protein essential for WNV replication, polyprotein processing, and immune evasion. It contains an N-terminal serine protease domain, requiring NS2B as a cofactor, and a C-terminal helicase domain. The NS2B–NS3 protease cleaves the viral polyprotein at multiple junctions (e.g., C-prM, NS2A-NS2B, NS2B-NS3, NS3-NS4A, NS4A-2K, NS4B-NS5), thereby releasing proteins required for replication and assembly (Erbel et al., 2006; Kuno and Chang, 2007; Chappell et al., 2008). Structural analyses show the protease domain adopts multiple conformations, supporting an induced-fit catalytic mechanism (Chappell et al., 2008; Robin et al., 2009).

The helicase domain exhibits NTPase and RTPase activities, unwinding dsRNA intermediates and removing the γ-phosphate during 5′ RNA capping (Chambers et al., 1990; Chernov et al., 2008; Roy et al., 2024). Unlike the protease, the helicase suppresses type I IFN signaling, with the NY99 strain showing enhanced immune antagonism (Fiacre et al., 2020). NS3 also interacts with viral proteins such as NS5 through defined surface “hotspots,” which are critical for efficient replication (Brand et al., 2024). Beyond enzymatic roles, NS3 contributes to virion budding, membrane reorganization, and apoptosis induction (Chambers et al., 1990; Chappell et al., 2008; Setoh et al., 2017; Kiemel et al., 2024). As disruption of either domain is lethal for replication, NS3 remains a prime antiviral drug target (Robin et al., 2009).

##### 2.3.2.5 Protein NS4A

This is a small, hydrophobic nonstructural protein essential for viral replication, ER membrane remodeling, and host cell modulation. It is anchored in the ER via internal hydrophobic domains, with its N-terminus exposed to the cytoplasm and the C-terminus embedded in the membrane. Topology is determined by proteolytic cleavages: the N-terminus by the viral NS2B-3 protease, and the NS4A-4B junction by a host signalase, ensuring proper orientation and function (Ambrose and Mackenzie, 2011). A conserved C-terminal tetrapeptide (^120^PEPE^123^) is critical for replication, as mutations impair RNA synthesis and NS2B-3 cleavage at the 2K region, which itself acts as a signal peptide for NS4B processing (Ambrose and Mackenzie, 2011; Ambrose and Mackenzie, 2015).

Functionally, NS4A integrates into the WNV RNA replication complex, forming homodimers and interacting with NS3, NS5, and NS2A (Ambrose and Mackenzie, 2011; Mikulasova et al., 2021). It acts as a cofactor for NS3 helicase, facilitating RNA unwinding and modulating ATPase activity through its hydrophilic N-terminal and acidic C-terminal motifs (Shiryaev et al., 2009). Together with NS4B, NS4A suppresses Akt-mTOR signaling and antagonises MDA5-mediated responses and the ISR (Ngueyen et al., 2019).

A cholesterol recognition motif in the N-terminus enables NS4A to remodel ER membranes and recruit proteins to cholesterol-rich domains, supporting replication complex biogenesis (Mikulasova et al., 2021). This drives the formation of virus-induced membrane structures and influences host signaling and immune evasion (Klaitong and Smith, 2021). Mutations in conserved residues reduce replication, protein stability, and membrane proliferation. Both NS4A and NS4B contribute to virulence in WNV and other orthoflaviviruses, including DENV, JEV, and YFV (Fiacre et al., 2023).

Protein NS4B: this is a small, hydrophobic ~27 kDa NS protein essential for viral replication, ER membrane remodeling, and immune evasion (Wicker et al., 2012; Wang et al., 2022). It is anchored in the ER and generated by sequential cleavage of the NS4A-2K-NS4B precursor: first by the viral NS2B/NS3 protease, then by a host signalase (Roosendaal et al., 2006; Miller et al., 2007; Wang et al., 2022). NS4B contains multiple transmembrane domains, is highly conserved among orthoflaviviruses, and shares up to 80% sequence similarity with JEV NS4B (Wang et al., 2022).

Although enzymatically inactive, NS4B is indispensable for RNA replication through interactions with other NS proteins. Its association with NS1 is critical for RNA synthesis, with compensatory mutations in one protein rescuing defects in the other (Youn et al., 2012; Wang et al., 2022). NS4B also binds the N-terminal region of NS3, enhancing helicase activity and RNA unwinding (Lu et al., 2021; Wang et al., 2022). Co-localization with NS4A in virus-induced structures suggests a role in replication organelle formation, while its 2K-dependent processing is necessary for generating ER-derived vesicles that host RNA replication (Kaufusi et al., 2014; Wu et al., 2017; Wang et al., 2022).

NS4B is a potent immune antagonist, suppressing IFN signaling by blocking RLR-induced TBK1 activation (Wu et al., 2017). Mutations, such as P38G, significantly reduce WNV virulence and neuroinvasiveness in mice (Wicker et al., 2012). It also disrupts mitochondrial dynamics and, with NS4A, inhibits the Akt-mTOR pathway, altering cellular homeostasis (Liang et al., 2016; Wang et al., 2022). Additionally, NS4B supports virion assembly by binding membranes and recruiting RNA into nucleocapsids. Owing to its multifunctional roles in replication, immune evasion, and assembly, NS4B represents a promising antiviral target (Wang et al., 2022).

##### 2.3.2.6 Protein NS5 (RNA-dependent RNA polymerase)

NS5 is the largest and most conserved orthoflaviviral protein, essential for viral replication and an attractive antiviral target (Goh et al., 2024). It replicates the positive-sense genome and synthesizes the negative strand. NS5 harbors two enzymatic domains: an N-terminal methyltransferase (MTase) that caps the viral RNA by methylating the 5’ GpppA structure at guanine-N7 and adenine-2′O (Saw et al., 2019), preventing immune recognition and enabling translation (Fajardo et al., 2020); and a C-terminal RNA-dependent RNA polymerase (RdRp) with a canonical closed-hand structure for genome replication (Jia & Gong, 2019). In addition, NS5 possesses a C-terminal PDZ-binding motif (PBM) that interacts with host PDZ domain proteins (e.g., TJP1, PARD3, ARHGAP21, SHANK2), and its disruption impairs WNV replication (Giraud et al., 2021).

Beyond replication, NS5 antagonizes type I IFN signaling. ZIKV NS5 blocks TBK1/IKKϵ activation (Lin et al., 2019; Lundberg et al., 2019) and mediates STAT2 proteasomal degradation via E3 ligases (Grant et al., 2016; Kumar et al., 2016; Ren et al., 2024), whereas WNV NS5 inhibits STAT1/2 transcriptional activity without inducing their degradation (Laurent-Rolle et al., 2010). In WNV-infected human cells, NS5 prevents STAT1/2 phosphorylation, while in mouse cells phosphorylation occurs but nuclear translocation is blocked (Pulit-Penaloza et al., 2012a; Pulit-Penaloza et al., 2012b). NS5 also disrupts SUMO1–STAT2 colocalization with PML, triggering PML degradation and impairing recruitment of the PAF1 complex to IFN-stimulated genes (Zhou et al., 2007; Ashour et al., 2009; Issur et al., 2009). Certain WNV strains, such as Kunjin, further exploit NS5 nuclear localization to repress host innate immune gene expression (López-Denman et al., 2021).

### 2.4 Post-translational modifications of viral proteins

Maturation and function of WNV proteins depend on a series of tightly regulated post-translational modifications (PTMs), including glycosylation, phosphorylation, ubiquitination, SUMOylation, acetylation, and glutathionylation (**Figure 2**).

E protein N-linked glycosylation plays an important role in proper folding, stability, and function, as well as in virulence, in both mammalian and mosquito hosts (Kumar et al., 2021). Glycosylation of the prM protein is also critical for the correct assembly and secretion of viral particles (Calvert et al., 2012). NS1, which requires N-linked glycosylation, is also essential for efficient secretion from infected cells (Fang et al., 2023).

Along with host factors such as protein kinase C or importin and HDM2, C protein is regulated by phosphorylation, which influences nucleocapsid assembly and viral replication (Bhuvanakantham et al., 2010; Cheong and Ng, 2011). Phosphorylation modifications are also produced on NS proteins, such as NS5. These modifications can alter the proteins’ enzymatic activities and their interactions with the host cell machinery, thereby impacting viral RNA synthesis (Boytz and Laurent-Rolle, 2024).

NS5 undergoes serine residue phosphorylation, which triggers its nuclear localization. Furthermore, NS5 requires post-translational modification by small ubiquitin-like modifier (SUMO) to regulate IFN-stimulated genes (ISGs) induced by IFN signaling. NS proteins, such as NS3 and NS5, may undergo acetylation and glutathionylation. These processes can regulate the proteins’ enzymatic activities and their interactions with viral RNA or host proteins. The viral serine protease NS3 is acetylated by the host factor KAT5. This acetylation modulates NS3’s RNA binding and helicase activities, and plays a crucial role in positively regulating viral replication (Serman et al., 2023).

The prM protein undergoes cleavage by host furin-like proteases in the trans-Golgi network, which is a necessary maturation step for producing infectious virions (Stoddard et al., 2015). This removes the N-terminal pr (pre) peptide and converts prM into the mature membrane (M) protein (Calvert et al., 2012). Once the virus is released into the neutral pH of the extracellular environment, the pr peptide dissociates, making the virion fusion-competent and infectious (Gollins and Porterfield, 1986; Smit et al., 2011; Pierson and Kielian, 2013; Sirohi and Kuhn, 2017; Kocabiyik et al., 2025). Uncleaved prM prevents the necessary conformational changes in E that are required for membrane fusion and cell entry (Nelson et al., 2008; Brinton, 2013; Slon Campos et al., 2018).

The NS5 protein contains a PDZ-binding motif (PBM) that interacts with host PDZ domain-containing proteins. Mutations in this motif reduce viral replication, which underscores the significance of precise PTMs for viral fitness and host interaction (Giraud et al., 2021).

## 3 Viral cycle and replication

**Figure 3:**
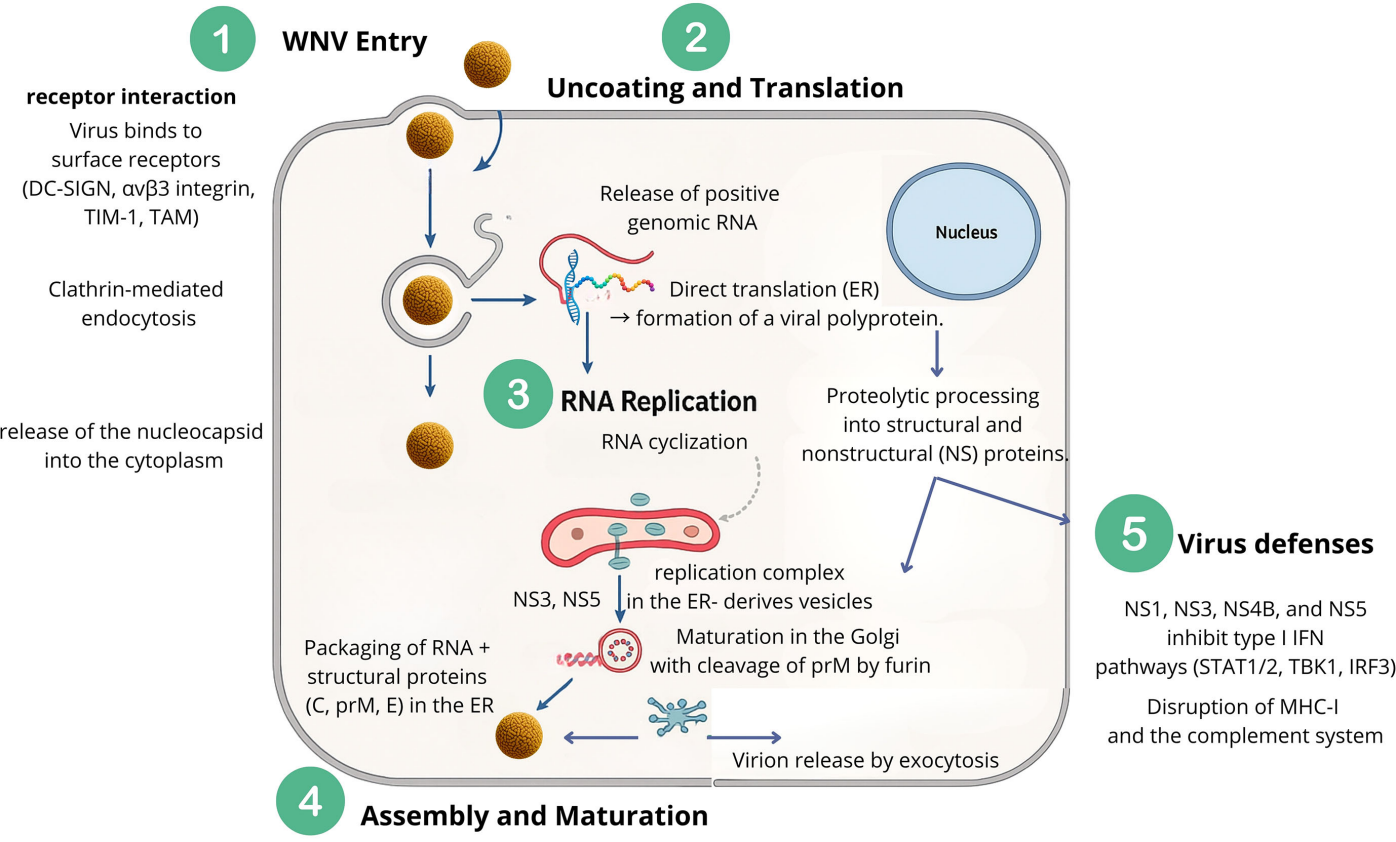
Replication cycle of WNV. **(1)** Viral entry occurs through receptor-mediated endocytosis, followed by **(2)** uncoating and release of genomic RNA. Translation in the endoplasmic reticulum (ER) produces a polyprotein processed into structural and non-structural proteins. **(3)** RNA cyclization and replication complexes form on ER-derived vesicles, enabling RNA synthesis and cyclization. **(4)** Assembly occurs in the ER with structural proteins (C, prM, E), followed by maturation in the Golgi via prM cleavage, and release by exocytosis. **(5)** Immune evasion by viral proteins (NS1, NS3, NS4B, NS5) antagonizes host immunity by blocking type I interferon, MHC-I, and complement responses.

Like many other orthoflaviviruses, WNV initiates infection through a series of crucial steps: entry into the host cell, interaction with receptors, and genome uncoating (**Figure 3**). Generally speaking, WNV viral cycle begins with entry into the host cell via receptor-mediated endocytosis, followed by fusion of the viral envelope with the endosomal membrane. The viral RNA genome is then released into the cytoplasm, where it serves directly as mRNA for the synthesis of viral proteins. Replication occurs in association with membranous structures derived from the endoplasmic reticulum, where a negative-sense RNA intermediate is synthesized and used as a template for producing new positive-sense genomes. Assembled viral particles bud into intracellular vesicles and are transported to the cell surface for release, enabling the infectious cycle to continue and facilitating the spread of WNV within the host organism. Each step of the WNV cycle will be discussed in detail in the following sections and summarized in **Figure 3**.

### 3.1 Virus entry, receptor interaction and uncoating.

Early WNV replication relies on the E protein, which mediates attachment, internalization via endocytosis, and genome release into the cytoplasm (Smit et al., 2011). In most mammalian cells, WNV enters primarily through clathrin-mediated endocytosis, rapidly localizing to early endosomes within five minutes post-entry; inhibition of clathrin or Rab5 impairs infection (Chu and Ng, 2004; Krishnan et al., 2007). Alternative pathways, such as lipid raft-mediated uptake in epithelial cells, exploit cholesterol-rich microdomains, illustrating viral adaptability (Medigeshi et al., 2008).

WNV engages multiple receptors to facilitate entry, including C-type lectins (DC-SIGN, DC-SIGNR), integrins (αvβ3), TIM family proteins (TIM-1), and TAM receptor tyrosine kinases (Tyro3, Axl, Mer), often through bridging molecules like Gas6 or Protein S, or via phosphatidylserine interactions (Chu and Ng, 2004; Davis et al., 2006; Lemke, 2013; Moller-Tank et al., 2014; Niu et al., 2018; Anwar et al., 2022). These interactions enable infection across diverse cell types, including neuronal, epithelial, immune, and mosquito cells.

Following endosomal fusion, the viral nucleocapsid is released and undergoes uncoating, facilitated by E protein conformational changes, ionic shifts, and cytosolic conditions, rendering the genome accessible for translation (Koschinski et al., 2003; Modis et al., 2004). Host restriction factors such as IFITMs can impede uncoating by modifying endosomal membranes, though WNV possesses countermeasures whose mechanisms remain under study (Gorman et al., 2016).

### 3.2 Replication

Translation of incoming positive-sense WNV RNA produces NS proteins essential for replication. Early in infection, the genome alternates between linear mRNA for translation and cyclized RNA for negative-strand synthesis, stabilized by 5’-3’ RNA interactions (Lo et al., 2003). Initial RNA synthesis is low, with negative-strand RNA remaining scarce throughout infection. NS protein accumulation induces ER membrane rearrangements, forming semioccluded replication complexes resistant to host restriction factors, where all NS proteins co-localize with dsRNA (Klema et al., 2015; Goh et al., 2024; Donaldson et al., 2025).

The RdRp domain of NS5 adopts a right-hand structure, initiating genome synthesis *de novo*, while NS3 helicase unwinds dsRNA intermediates; NS5–NS3 interactions are critical for replication (Tay et al., 2015; Ferrero et al., 2018). Genome cyclization via 5’–3’ UTR interactions is essential for negative-strand synthesis and overall viral gene expression; deletion of UTR structures is lethal (Holden and Harris, 2004; Anthony et al., 2009; Villordo et al., 2010; Brinton, 2013). Newly synthesized positive-strand RNA is then released for translation, replication, or assembly into virions on ER membranes (Goh et al., 2024; Donaldson et al., 2025).

## 4 Epidemiological cycle of WNV

**Figure 4:**
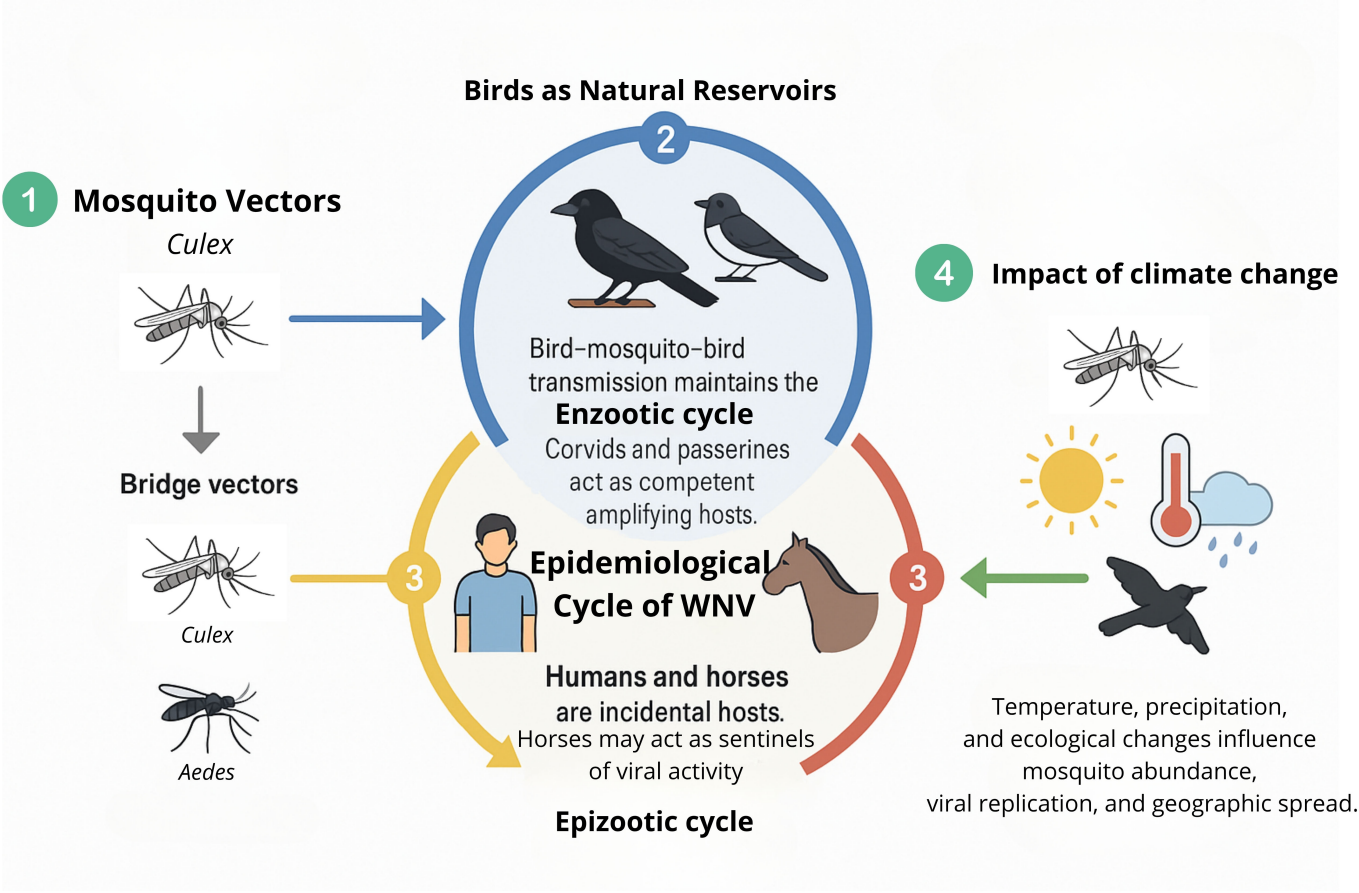
Epidemiological cycle of WNV. **(1)** Culex mosquitoes act as primary vectors; bridge vectors (Culex, Aedes) transmit to incidental hosts. **(2)** Birds maintain the enzootic cycle, with corvids and passerines as amplifying hosts. **(3)** Humans and horses are dead-end hosts; horses may serve as sentinels. **(4)** Climate change affects mosquito abundance, viral replication, and geographic spread.

WNV persists in nature via two distinct transmission cycles. The enzootic cycle is characterized by continuous viral circulation between avian hosts and ornithophilic mosquito vectors, primarily Culex species. In contrast, the epizootic cycle involves incidental, or “dead-end,” infections in mammals such as humans and horses, which do not contribute substantially to further viral transmission. The specific features of each cycle will be detailed in the subsequent sections (**Figure 4**).

### 4.1 Birds as natural reservoirs

Wild birds represent the primary natural reservoirs of WNV. Among these, hatchlings are particularly important due to their ability to develop high levels of viremia sustained over several days (typically 2 to 7 days), which supports efficient virus amplification and transmission (Grubaugh et al., 2015). Galliform birds exhibit low susceptibility; although they can develop transient viremia and mount antibody responses, they rarely show clinical signs and are not significant contributors to virus persistence.

Corvids and passerines, such as crows, jays, magpies, sparrows, and finches, are highly competent reservoirs of WNV in Europe. They frequently survive infection and maintain viremia levels high enough to infect feeding mosquitoes. This perpetuates the viral transmission cycle. In contrast, raptors generally fail to develop high viremia and thus rarely infect mosquitoes, making them poor reservoirs from an epidemiological standpoint. Nevertheless, raptors are highly susceptible to WNV, and infections often result in significant morbidity and mortality (David et al., 2007). Due to these severe outcomes, raptor deaths reliably signal local viral activity and serve as sensitive sentinels. This sentinel role is crucial because raptors’ conspicuous susceptibility increases the detection of WNV circulation in an area. This indirectly aids public health and surveillance efforts and plays a central—though indirect—role in supporting the continued enzootic transmission cycle across.

Notably, WNV exhibits a capacity for rapid adaptation to both invertebrate vectors and vertebrate hosts, potentially allowing the virus to exploit new ecological niches and establish novel transmission foci (Brault, 2009). Migratory birds are key facilitators of both local and long-range dissemination of the virus, often bridging distinct geographic regions. In temperate climates, peak viral activity in birds typically occurs from mid-summer to early autumn, with human and equine cases following shortly thereafter.

### 4.2 Incidental hosts and epizootic transmission

During the epizootic cycle, WNV infects incidental hosts, primarily humans and horses. Other mammalian species, such as cats, dogs, sheep, goats, squirrels, skunks, and rabbits, can also become infected through mosquito bites, but they are generally considered dead-end hosts due to their inability to sustain viremia at levels sufficient to infect mosquitoes (Kramer and Bernard, 2001). Thus, they are of limited epidemiological relevance in the virus transmission cycle.

Neither humans nor horses develop viremia levels high enough to propagate further transmission (Schwarz and Long, 2023). Importantly, horses can act as sentinel species; the appearance of clinical signs in equine populations often serves as an early indicator of WNV activity in a given area (García-Carrasco et al., 2023). This sentinel function provides valuable lead time for public health and veterinary authorities to initiate vector control and preventive measures (Magallanes et al., 2023).

### 4.3 Mosquito vectors

WNV is primarily transmitted by ornithophilic mosquitoes of the genus Culex, which are responsible for sustaining the enzootic cycle in nature (Schwarz and Long, 2023). Female mosquitoes of Culex spp. play significant roles in viral transmission through their blood-feeding activity. Although WNV has occasionally been isolated from other arthropods such as ticks, the absence of effective transstadial transmission excludes them from meaningful epidemiological relevance (Lawrie et al., 2004).

Multiple Culex species are competent vectors of WNV in diverse ecological contexts. In the US, *Culex pipiens*, *Culex quinquefasciatus*, and *Culex tarsalis* have been identified as major vectors (Diaz-Badillo et al., 2011). In Europe, the main vectors are *Culex perexiguus*, *Culex pipiens*, *Culex modestus*, and *Culex laticinctus*. In Africa, particularly sub-Saharan regions and the interior of South Africa, *Culex univittatus* is considered the principal vector, while *Culex neavei* predominates in the Natal lowlands South Africa (Jupp and McIntosh, 1970; Jupp et al., 1986). In Asia, *Culex tritaeniorhynchus* is an established vector in South and East Asia, along with other proposed species such as *Culex vishnui*, *Culex pseudovishnui*, *Culex quinquefasciatus*, and *Culex gelidus* (Ahmed et al., 1979; Natasha et al., 2023).

Vector competence in mosquitoes requires the presence of specific midgut receptors that facilitate viral entry and replication, followed by viral dissemination to the salivary glands to enable subsequent transmission to vertebrate hosts. Quantifying viral load in mosquitoes is crucial for evaluating vector competence and transmission risk (Grubaugh and Ebel, 2016). Reported viral RNA levels range from 10^4^ to 10^10^ copies per mosquito, with peak infectivity occurring during the transmission phase, when the virus reaches the salivary glands. This measurement provides a useful indicator of the mosquito’s potential to transmit WNV to vertebrate hosts and supports risk-based surveillance efforts. WNV is capable of both transstadial and transovarial transmission within mosquitoes, mechanisms that facilitate viral persistence across seasons. The seasonal dynamics of WNV infection are closely aligned with vector activity (Ruiz-López et al., 2023). At the end of the summer, additional mosquito genera such as *Aedes* and *Ochlerotatus* may serve as bridge vectors. These species feed on both birds and mammals, facilitating spillover from the enzootic to the epizootic cycle. Furthermore, hybridization events involving *Culex pipiens* and closely related taxa have been associated with shifts in feeding behavior, including a reduction in exclusive ornithophily, which increases transmission potential to humans and other mammals. Recent genomic studies conducted in southern Spain on Culex-borne WNV Lineage 1 strains are beginning to study mosquito genes potentially involved in viral transmission and host adaptation.

### 4.4 Impact of climate change

WNV is one of the most rapidly spreading orthoflaviviruses in recent decades. Since the 1990s, its epidemiology has shifted due to increased globalization, international trade, and human travel (Kramer et al., 2019). Major outbreaks in the U.S. (1999), Dallas, Texas (2012), Maricopa County, Arizona (2021) (Chung et al., 2013; Kretschmer, 2023), and Europe (2018) highlighted its ability to cause severe encephalitis and establish itself as a significant global vector-borne pathogen (Nash et al., 2001; Camp and Nowotny, 2020; Pietsch et al., 2020). Following its introduction to America, research has enhanced our understanding of host-vector-pathogen-environment interactions, including the influence of climate on virus proliferation and transmission and the challenges of preventing its establishment in ecosystems with abundant wildlife reservoirs (Kramer et al., 2019). Although arboviruses tolerate a broad temperature range, climate variability affects viral structure, vector populations, and transmission dynamics, potentially driving viral evolution and outbreak severity (Ciota and Keyel, 2019; Bellone and Failloux, 2020). Warmer temperatures have been linked to increased WNV strain diversity and enhanced transmission potential (Fay et al., 2025).

Mosquitoes are ectothermic arthropods; therefore, their physiology and reproductive cycles are temperature-dependent (Bellone and Failloux, 2020). Rising temperatures accelerate mosquito development and shorten the extrinsic incubation period, i.e. the time required for the virus to reach the mosquito’s salivary glands after ingestion, thus increasing transmission probability (Sardelis et al., 2001; Reisen et al., 2006; Samuel et al., 2016; Reinhold et al., 2018). These factors also influence habitat expansion and avian migration patterns, enhancing virus spread (Kunkel et al., 2006; Jia et al., 2007; Mordecai et al., 2019). Humidity and precipitation also impact mosquito populations and WNV transmission. Increased rainfall can create new breeding sites, while droughts may lead to water storage practices that unintentionally support mosquito proliferation. However, the impact of humidity is less clearly understood (Becker, 2008; Reiter, 2008; Bellone and Failloux, 2020; Drwiega et al., 2024).

Global mobility and urbanization further complicate disease dynamics, facilitating mosquito introduction into favorable climates and altering transmission patterns (Patz et al., 2000). Despite growing evidence on climate-driven changes in WNV transmission, a comprehensive response plan remains absent. There is a need to gather solid evidence to support future decision-making and strategies that will allow us to adapt and prepare public health means (Wang et al., 2024).

## 5 Host defenses

### 5.1 Mosquito host defenses

Given the importance of the epidemiological cycle of WNV, mosquitoes possess a range of innate immune defenses to counter WNV infection. Their primary antiviral strategies involve several conserved immune pathways, such as RNA interference (RNAi), and the Toll, Imd, and JAK-STAT signaling cascades. Of these, the small interfering RNA (siRNA) system is especially crucial, as it can detect and degrade viral RNA inside mosquito cells. Disruption of this pathway leads to higher viral replication and transmission efficiency, underlining its protective role. Additionally, the piwi-interacting RNA (piRNA) pathway also helps impede virus proliferation, while specific microRNAs may limit West Nile infection in certain mosquito species (Cheng et al., 2016).

These molecular defenses enable mosquitoes to tolerate persistent, lifelong viral infections—a trait vital to their survival as vectors. The interplay among siRNA, piRNA, and microRNA pathways demonstrates a sophisticated multi-tiered response; for example, piRNA-like virus-specific small RNAs have been found in mosquitoes infected with various arboviruses, and experimental suppression of piRNA machinery increases viral replication. Differential microRNA expression has been documented in mosquitoes infected with WNV, and some miRNAs in Aedes aegypti appear to restrict viral infection, showing species-specific adaptations (Cheng et al., 2016).

While these internal defenses do not fully clear the virus, they regulate viral loads and confer mosquito resilience, allowing the insect to survive and transmit WNV without succumbing to the disease. Such mechanisms are far from understood and will deserve further investigation in the future. However, the presence of these antiviral mechanisms highlights mosquitoes’ evolutionary adaptation to coexist with—and spread—pathogenic viruses. Ongoing research aims to exploit these pathways for novel vector control strategies, potentially reducing transmission of WNV and other mosquito-borne diseases (Cheng et al., 2016).

Despite the above mechanisms, WNV can still establish high viral loads in mosquito salivary glands due to viral countermeasures that suppress or evade the mosquito’s immune response, enabling efficient transmission without harming the mosquito itself. Gaps in knowledge persist regarding the full spectrum of antiviral factors active in mosquitoes and the host–vector–virus interactions, particularly as ecological changes—such as those driven by climate and land use—alter mosquito distribution and virus epidemiology. Future perspectives involve better elucidation of mosquito immune strategies, development of sophisticated models to predict WNV transmission under environmental change, and integration of these findings into vector control and surveillance systems to mitigate the risk of human infection (Martin and Nisole, 2020; Rosenkrantz, 2022; Erazo et al., 2024).

### 5.2 Mammalian host defenses

WNV infection triggers a robust cellular immune response involving both innate and adaptive mechanisms in the mammalian host. On the one hand, pattern recognition receptors (PRRs) are innate immune sensors critical for the early detection of WNV infection, triggering of antiviral immunity. PRRs, including Toll-like receptors (TLRs), RIG-I-like receptors (RLRs) such as RIG-I and MDA5, and NOD-like receptors (NLRs), recognize viral components—especially viral RNA—immediately after WNV entry into host cells, orchestrating rapid innate responses to contain the virus (Saiz et al., 2021; Behari et al., 2024). Upon WNV infection, PRRs detect pathogen-associated molecular patterns (PAMPs) such as viral RNA. Key PRRs involved include TLR3, TLR7, RIG-I, and MDA5. Activation of these receptors triggers downstream signaling pathways involving adaptor molecules like IPS-1 (also known as MAVS) and MyD88, which in turn activate transcription factors such as IRF3 and IRF7. This results in robust production of type I interferons (IFN-α/β) and pro-inflammatory cytokines that limit viral replication and spread (Suthar et al., 2010; Saiz et al., 2021; Behari et al., 2024). Experimental studies with knockout mice deficient in specific PRRs or their signaling adaptors show markedly increased susceptibility to WNV infection, higher viral burdens, and increased mortality. Such findings highlight the essential role of PRRs in initiating and shaping effective innate and downstream adaptive immune responses. Notably, PRR signaling via RIG-I, MDA5, TLR3, and TLR7 is necessary for controlling viral replication, restricting neuroinvasion, and ensuring quality antibody responses (Suthar et al., 2010; O’Ketch et al., 2020; Saiz et al., 2021).

Recent progress has been made in identifying specific ISGs that restrict WNV infection. Systematic investigations employing ectopic gene expression and short hairpin RNA (shRNA) screening approaches have revealed novel genes and gene families with antiviral activity against flaviviruses, including WNV. Upon infection, recognition of viral components by pattern recognition receptors, such as Toll-like receptor 3 (TLR3) and RIG-I-like receptors, activate signaling pathways that lead to the production of pro-inflammatory cytokines (e.g., IL-6 and TNF-α) and type I IFN. Importantly, IPS-1, the central adaptor protein to RIG-I-like receptor (RLR) signaling, plays an important role in establishing adaptive immunity through an innate/adaptive interface that elicits effective antibody responses and controls the expansion of regulatory T cells. These responses help recruit immune cells—such as monocytes, macrophages, and natural killer (NK) cells—to sites of infection, initiating antiviral defense and limiting early viral spread. Cocultures of dendritic and NK cells revealed that RLR and type I IFN signaling pathways are essential in promoting NK cell activation during WNV infection. Combined RLR- and type I IFN-dependent signaling programs drive specific antiviral effector gene expression and programs NK cell responses that, together, serve to restrict WNV tissue tropism; however most of the results are coming from mice model analysis (Suthar et al., 2010; Cho and Diamond, 2012; Suthar et al., 2013a).

Macrophages are central players in this process. They can directly clear WNV through phagocytosis and production of reactive oxygen species, as well as present WNV antigens to B and T lymphocytes to promote adaptive immunity. NK cells also exert cytolytic, polyfunctional response to WNV characterized by cytolytic activity, cytokine and chemokine secretion. This is associated with downregulation of activating NK cell receptors and upregulation of NK cell activating ligands for NKG2D. Meanwhile, dendritic cells (DCs) activate T cells by presenting viral antigens, orchestrating downstream adaptive responses (Diamond and Gale, 2012; Yao et al., 2017).

The adaptive immune response is characterized by the mobilization of CD8+ cytotoxic T cells and CD4+ helper T cells, which are critical for clearing the infection from neuronal tissues and sustaining overall immune activity. CD8+ T cells are recruited into the central nervous system (CNS) by chemokines such as CXCL10 and eliminate infected neurons through Fas-mediated cytolysis. The effectiveness and regulation of the T-cell response are crucial—while insufficient activity results in poor viral clearance, an overactive response may cause immunopathology and neuronal injury (Bhuvanakantham and Ng, 2009; Cho and Diamond, 2012).

WNV has evolved several strategies to evade or suppress these cellular immune defenses. WNV can dampen DC activation and inhibit the expression of costimulatory molecules and proinflammatory cytokines, leading to impaired priming and proliferation of virus-specific T cells. Such viral interference weakens both the magnitude and quality of T cell immunity, potentially resulting in more severe disease. Additionally, WNV can manipulate innate cytokine signaling—such as downregulating activating NK cell receptors—to escape early immune detection and destruction, thereby enhancing its replication and pathogenesis (Yao et al., 2017; Zimmerman et al., 2019).

The humoral immune response is a key component in protection against WNV infection. Upon exposure to WNV, B cells rapidly produce IgM antibodies, typically detectable within 3–8 days after symptom onset, and these can persist for months—even longer in some cases. IgG antibodies appear later and, although their levels may slowly decline over the years, studies show that many individuals maintain neutralizing antibody titers for up to five years post-infection. Both IgM and IgG play crucial roles in controlling viremia and preventing viral dissemination into the central nervous system. Neutralizing antibodies, targeting the viral envelope (E) protein—especially epitopes on DIII—are particularly potent. These antibodies block viral entry and fusion, thus halting the infectious cycle (Diamond et al., 2008; Throsby et al., 2009; Chabierski et al., 2013; Carson et al., 2014; CDC, 2024).

While animal studies suggest a rapid and robust humoral response, human responses are notable for prolonged IgM persistence and slower IgG onset. The majority of antibody binding in humans’ targets epitopes that are not always associated with optimal neutralization, meaning some immune responses may be directed toward weakly neutralizing regions of the E protein rather than strongly protective sites. Nonetheless, passive transfer experiments and therapeutic trials with monoclonal antibodies have demonstrated that sufficient antibody levels, particularly those with high affinity for critical viral epitopes, can be highly effective at neutralizing the virus and providing lasting protection—even when administered days after infection. Overall, humoral immunity is pivotal for both recovery and long-term defense against WNV, making it a central focus for vaccine development and post-exposure therapies (Eisenstein, 2005; Diamond et al., 2008; Carson et al., 2014). Additionally, pre-exposure prophylaxis strategies, particularly vaccine candidates (e.g., phase I trials of inactivated or DNA vaccines) and monoclonal antibodies administered prior to infection, are being actively explored as complementary approaches to enhance protection in high-risk populations (Martin et al., 2007; Cendejas and Goodman, 2024; Calvert et al., 2025).

Mammalian host defenses are crucial for restricting WNV infection, limiting viral replication, and preventing serious outcomes such as neuroinvasive disease. Interferon pathways, including type I interferons and interferon-stimulated genes, play a central role in initiating an antiviral state and orchestrating adaptive immunity, while other mechanisms—such as the OAS/RNase L system, apoptosis, and antibody-mediated responses—contribute additional layers of protection. Despite these advances, significant knowledge gaps remain, including incomplete understanding of how different immune pathways interact, why certain individuals develop severe disease, and the mechanisms underlying long-lasting immunity or immune evasion by the virus. Future challenges include the development and validation of effective vaccines and antivirals for humans, deeper experimental investigation of immune responses in relevant models, and addressing the unpredictable clinical spectrum of WNV infection as the virus expands into new geographical regions due to climate change (Ahlers and Goodman, 2018; Habarugira et al., 2020; Kocabiyik et al., 2025).

## 6 Intra-host genetic variability and quasispecies

Phylogenetically, WNV is divided into seven genetic lineages, of which lineages 1 and 2 are the most clinically relevant. Lineage 1 strains, associated with high virulence, have been implicated in major human outbreaks and can be subdivided into three sub lineages: L1a, L1b, and L1c. The WNV NY99 strain, which caused the initial US outbreak and exhibits high virulence, belongs to sub lineage L1a (Tajima et al., 2024). L1b and L1c exhibit distinct epidemiological and host-specific features. Lineage 1 includes the most pathogenic strains and circulates in regions including southern Spain, Africa, the Middle East, India, America and Australia (Tajima et al., 2024). Lineage 2 strains are found in South Africa, Madagascar, and parts of Europe (Aguilera-Sepúlveda et al., 2024; Maroco et al., 2025). Recently, West Nile virus (WNV) lineage 3 was detected in a human case in Nebraska, demonstrating its clinical relevance beyond its previous detection in avian and mosquito populations, and highlighting its potential for human spillover (Davis et al., 2023). In contrast, lineage 1 in North America is expanding rapidly, while outbreaks in southern Europe tend to be localized and non-recurrent. (Silverj et al., 2024). L1b strains have been linked to a greater burden of human disease in the rest of the American continent, while L1c strains demonstrate heightened pathogenicity in certain avian species compared to European analogs. These differences may reflect intrinsic viral genetic determinants or environmental factors that influence transmission dynamics.

**Figure 5:**
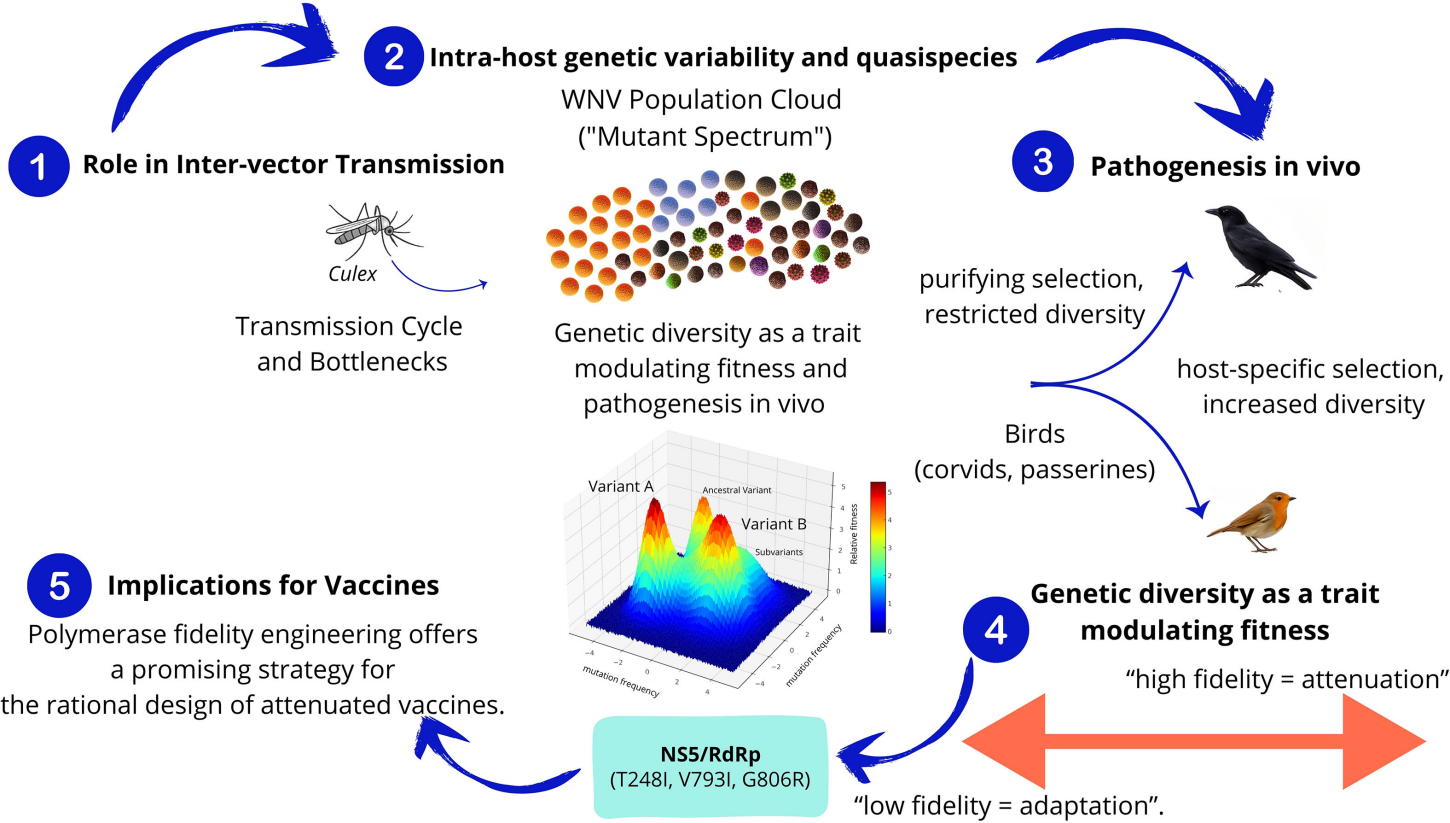
Intra-host genetic variability and quasispecies. **(1)** Role in Inter-vector Transmission. Transmission between hosts imposes genetic bottlenecks that constrain diversity, which is later restored during replication and adaptation. **(2)** Intra-host genetic variability and quasispecies. WNV exists as a mutant spectrum or quasispecies composed of genetically related variants subjected to mutation, selection, and genetic drift. **(3)** Pathogenesis *in vivo*. Selective pressures differ between mosquito and avian hosts, shaping the quasispecies structure in a host-specific manner. **(4)** Genetic diversity as a trait modulating Fitness. RdRp fidelity modulates viral diversity and fitness. Mutations increasing fidelity led to attenuation and reduced transmissibility. NS5/RdRp protein box: Shows specific mutations (T248I, V793I, G806R). **(5)** Implications for Vaccines. Polymerase fidelity engineering offers a promising strategy for the rational design of attenuated vaccines.

Genomic analyses have characterized WNV as a population of genetically distinct variants that act collectively as a unit of natural selection (Jerzak et al., 2005; Van Slyke et al., 2012) (**Figure 5**). WNV displays remarkable genomic variability, with an estimated rate of approximately five nucleotide substitutions per genome per year (Tang et al., 2008), corresponding to a mutation rate 10^5^ to 10^8^ times higher than that of its host’s genomic DNA (Holland et al., 1982; Domingo and Holland, 1997).

### 6.1 WNV quasispecies and its role in inter-vector transmission

WNV high mutation rate results from low copying fidelity in RNA-dependent RNA polymerases (Elena and Sanjuán, 2005), endowing WNV with the ability to rapidly adapt to different organs, tissues, and cells, and to switch hosts across its transmission cycles (Wagner et al., 2008; Sim et al., 2015).

These findings align with the quasispecies concept proposed by M. Eigen in 1971 (Eigen and Schuster, 1978) and later confirmed for RNA viruses (Domingo and Holland, 1997; Lauring and Andino, 2010; Rozen-Gagnon et al., 2014). Viral quasispecies are defined as complex distributions of closely related variant genomes subjected to genetic variability, competition, selection, and genetic drift (Domingo et al., 2012; Domingo and Perales, 2019). Currently, few studies have analyzed WNV quasispecies structure and intra-population genetic changes relative to consensus sequences. The role of mutation-selection balance within intra- and inter-host variants has often been underestimated. However, this equilibrium is essential to quasispecies dynamics, ensuring viral adaptation to new hosts and tissue tropism (Kortenhoeven et al., 2015). Additionally, the route of inoculation may significantly influence quasispecies dynamics, as primary replication sites following subcutaneous (SC) versus intracerebral (IC) inoculation involve fundamentally different cellular populations (Dridi et al., 2015).

This variable population structure enables WNV to maintain its interspecies transmission cycle (**Figure 5**). Although the complexity of WNV populations does not appear to decrease significantly during the extrinsic incubation period of mosquitoes (Brackney et al., 2011). Genetic variant populations are mainly constrained within mosquito vectors (Brackney et al., 2015), although significant genome remodeling dependent on mosquito species has been observed (Fitzmeyer et al., 2023). Studies have shown that the plasticity of the virus’s genomic variant spectrum is particularly important for arboviruses circulating among taxonomically divergent hosts. This suggests that consensus sequences alone do not represent overall genetic diversity, making consideration of minority genotypes essential for an accurate and complete understanding of arbovirus evolution and adaptation within and between hosts (Caldwell et al., 2020). Certain avian species, particularly passerines like corvids, also act as key amplifying hosts. Notably, next-generation sequencing (NGS) analysis have experimentally demonstrated significant diversification of WNV populations within corvid hosts, diverging from the original inoculum structure (Dridi et al., 2015). This suggests that diversification is driven by host-specific selective pressures.

The “trade-off hypothesis” commonly posits that slower evolutionary rates in arboviruses result from the biological necessity of alternating replication in two taxonomically divergent hosts (vertebrates and arthropods). According to this model, viral fitness is reduced in each host compared to single-host viruses capable of specialization. Evidence supports that WNV adaptation is better predicted in avian environments than in mosquito vectors, suggesting that viral expansion cycles in mosquitoes are followed by purifying selection in birds (Deardorff et al., 2011). Significant reductions in sequence diversity during transmission events are considered genetic bottlenecks, with diversity restoration occurring after establishment in new tissues (Grubaugh et al., 2016). Persistent replication and population bottlenecks, characterized by the limited transmission of founder viral sequences to new hosts, are critical (Stoddard et al., 2015). Host switching necessitates adaptation within the mutant spectrum, selecting sequences capable of efficient infection in new hosts, with restored sequence diversity observed upon mosquito reinfection (Ciota et al., 2007; Brackney et al., 2015).

Previous studies indicate that WNV and other arboviruses undergo strong purifying selection (Woelk and Holmes, 2002; Twiddy and Holmes, 2003; Jerzak et al., 2005). Thus, understanding the intra-host primary genome sequence variation and its secondary RNA structures—critical regulators of genome cyclization, replication, and translation—is essential (Scroggs et al., 2019). These structures are vital for viral replication, translation, and microRNA binding, influencing antiviral therapy efficacy. Moreover, mosquito species with broader feeding preferences may enhance cross-species viral transmission, contributing to human outbreaks (Brault et al., 2002; Briese and Bernard, 2005). Vector competence significantly influences WNV evolutionary rates and quasispecies population dynamics (Fitzmeyer et al., 2023). Population structure is shaped by selective and stochastic processes, including transmission bottlenecks that affect viral infectivity. A detailed understanding of intra- and inter-host population dynamics is imperative for effective prevention strategies. Prior evidence suggests that the initial viral population’s composition critically determines infection maintenance (Ciota et al., 2012; McCrone et al., 2018).

Competent vectors enhance the probability of minority variants establishing infection in new hosts, altering quasispecies structure non-selectively (**Figure 5**). The growing epidemiological significance of WNV highlights the need for comprehensive studies on circulating viral variants to predict disease emergence and improve countermeasures. NGS studies of WNV lineage 2 demonstrate that minority variants maintain a stable mutation-selection balance, facilitating membrane association with host cells (Kortenhoeven et al., 2015). The increasing frequency of WNV outbreaks and the absence of an effective vaccine emphasize the urgency of understanding its quasispecies-driven mechanisms of cross-species transmission. Application of NGS technologies to study WNV quasispecies dynamics in arthropod vectors and avian hosts will be pivotal in elucidating the mechanisms of WNV endemization and pathogenic evolution.

### 6.2 Genetic diversity as a trait modulating fitness and pathogenesis *in vivo*

Several lines of study support that RNA viruses require vast genomic diversity, not only to evolve and adapt to the environment, but to successfully infect their natural hosts. Either increases or decreases in diversity cause virus attenuation, suggesting that polymerase fidelity is a master regulator of virus phenotype, and thus is finely tuned to reach the optimal degree of variation during infection (Vignuzzi et al., 2006; Van Slyke et al., 2015; Arias et al., 2016). Possibly the most paradigmatic example of the impact of restricted diversity on virus pathogenesis was obtained by Vignuzzi and colleagues who showed that a high-fidelity virus was completely attenuated *in vivo*, inspiring a novel line of research based on altering fidelity in the design of live vaccines (Vignuzzi et al., 2006; Vignuzzi et al., 2008). Further observations hint at a possible connection between fidelity and the emergence of variants with epidemiological success (Cheung et al., 2014; Andino and Domingo, 2015; Arias et al., 2016).

Similar to other virus polymerases, orthoflavivirus RdRp is highly error-prone, leading to elevated mutation rates during genome replication (Caldwell et al., 2022). Whilst orthoflavivirus remain one of the biggest threats to global health, there are limited studies investigating the fidelity of their polymerases during replication, and its manipulation to the design of attenuated variants. A high-fidelity WNV variant was isolated during adaptation to ribavirin, a mutagenic nucleoside eliciting increased mutation frequencies in the orthoflaviviruses (Van Slyke et al., 2015; Bassi et al., 2018). This variant contained three amino acid replacements in NS5; T248I, in the MTase domain, which caused decreased fidelity, and V793I and G806R in the thumb subdomain of the RdRp region, leading to increases in fidelity. All three mutations combined resulted in reduced error rates during RNA synthesis, and hence higher fidelity, in line with other studies on laboratory-driven virus adaptation to mutagens. When these three mutations were introduced into the infectious clone, the resulting WNV variant exhibited restricted replication during infection in mosquitoes, suggesting that restricting viral genetic diversity may also affect orthoflavivirus fitness *in vivo* (Van Slyke et al., 2015). In addition to WNV, polymerase fidelity checkpoints have also been identified in St Louis encephalitis virus (SLEV) NS5 protein. Likewise increases in SLEV fidelity also lead to reduced genetic infectivity, and hence limited virus infectivity in mosquito cells (Caldwell et al., 2022). Additional evidence supporting a connection between fidelity and virulence come from studies on YFV 17D vaccine. In the attenuation process, during sequential passages of virulent YFV in chick embryo cells, a high-fidelity polymerase variant was naturally selected, further supporting that reduced diversity restricts orthoflavivirus infectivity in the host (Davis et al., 2019). All these studies are hinting at quasispecies diversity as a major regulator of orthoflavivirus infectivity and pathogenesis, and therefore it appears to be precisely regulated by RdRp fidelity. Consequently, alterations in copying fidelity can be further investigated to the design of new or improved vaccines against WNV and other orthoflaviviruses (**Figure 5**).

## 7 West Nile fever

**Figure 6:**
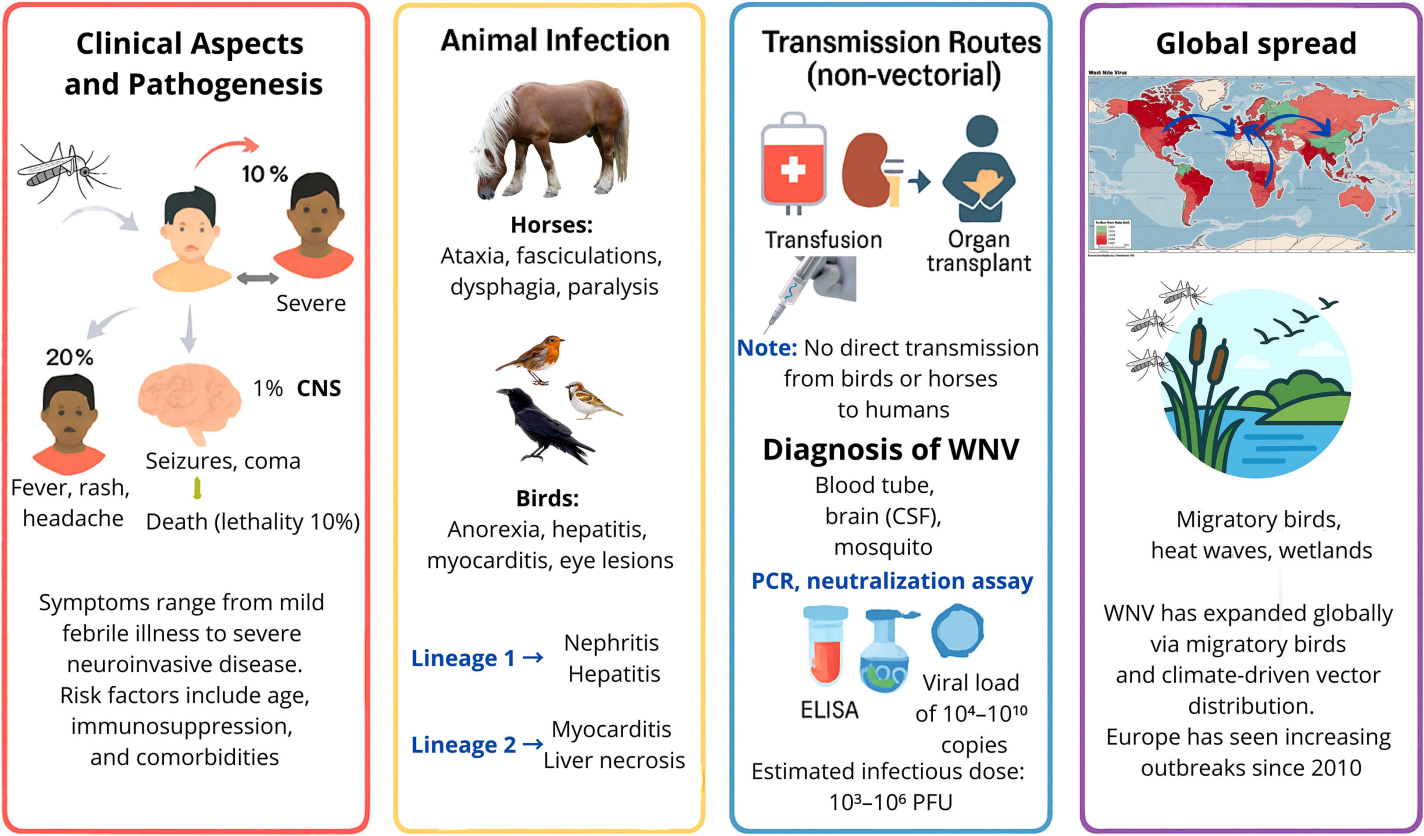
Infographic on West Nile Virus (WNV). Clinical aspects, animal infection, transmission routes, and the global spread of WNV. [Red right panel] Human disease ranges from mild febrile illness to severe neuroinvasive forms, influenced by age, immunosuppression, and comorbidities. [Yellow panel] In horses, infection causes neurological signs; in birds, pathology varies by lineage. [Blue panel up] Non-vectorial transmission occurs via transfusion or organ transplantation; [Blue panel down] diagnosis includes PCR, neutralization assays, and ELISA. [Violet panel] Global spread is linked to migratory birds and climate-driven vector expansion, with increasing outbreaks in Europe since 2010.

Every year, thousands of people are infected with WNV globally, but the disease often goes underreported because about 80% of infected people show few or no symptoms, according to the World Health Organization (WHO 2017). Approximately 20% of infected individuals are symptomatic, and 1% develop neuroinvasive disease, with a 10% fatality rate among severe cases (Clark and Schaefer, 2025). For example, recent data from Europe show hundreds of annual cases: in 2023 there were 709 cases and 67 deaths, and 1,116 cases in 2022 (Epidemiological update: West Nile virus transmission season in Europe, 2023, 2024). Clinical aspects, animal infections, transmission routes and global spreading are summarized in the following sections and in **Figure 6**.

### 7.1 Clinical aspects and pathogenesis

The incubation period for WNV in humans ranges from 3 to 14 days post mosquito bite. Initial viral replication occurs locally and in draining lymph nodes, followed by viremia that spreads the virus to the mononuclear phagocyte system (MPS) (Clark and Schaefer, 2025). If viremia persists, WNV can cross the BBB, with potential invasion of the CNS, infecting neurons and glial cells, particularly in the thalamus and spinal cord, leading to perivascular inflammation, neuronal necrosis, and microglial nodules (Samuel et al., 2016).

The clinical presentation of human cases can range from mild symptoms such as fever, headache, rash, and lymphadenopathy to severe neuroinvasive disease including meningitis, encephalitis, and acute flaccid paralysis (Petersen et al., 2013; Roberts et al., 2024). Older adults, immunocompromised individuals, and patients receiving B-cell depleting therapies are more likely to experience severe disease. These individuals exhibit impaired humoral responses and prolonged viremia (Kapadia et al., 2023), as well as comorbidities (Bampali et al., 2022). Central nervous system (CNS) involvement can present with seizures, disorientation, coma, and occasionally death (Sejvar, 2014). Rare complications include poliomyelitis-like syndromes, myocarditis, and uveitis, which complicate diagnosis (Habeb et al).

The immune response is pivotal in controlling infection. Robust humoral immunity, particularly neutralizing antibodies targeting critical epitopes of the E protein, correlates with recovery and protection, while suboptimal or delayed responses can exacerbate disease severity (Eisenstein, 2005; Diamond et al., 2008; Carson et al., 2014). Cellular immunity, including T-cell-mediated responses, also contributes to viral clearance and long-term protection.

Lineage-specific differences influence pathogenesis. Lineage 1 infections typically involve nephritis, hepatitis, and splenic lymphoid depletion, whereas lineage 2 is more frequently associated with myocardial necrosis and hepatic alterations (Williams et al., 2014; Venter et al., 2017). Birds and equines demonstrate species-specific clinical signs: raptors show severe neurological lesions and viral persistence in the CNS, while equines may present with ataxia, facial muscle twitching, dysphagia, paralysis, and in severe cases, coma or death (Angenvoort et al., 2013; Gamino and Höfle, 2013; Venter et al., 2017; Paré and Moore, 2018; Fehér et al., 2022).

Transmission primarily occurs via *Culex* spp. mosquitoes, though alternative routes, such as transfusions, organ transplantation, and vertical transmission, have been documented (Gould and Fikrig, 2004; Hayes et al., 2005). There has been no confirmed direct transmission from birds or horses to humans. Mechanistically, WNV neuropathogenesis involves a complex interplay of viral replication, host immune responses, and disruption of CNS homeostasis (Gern et al., 2024). Viral entry into the CNS is facilitated by endothelial cell infection and damage, increased blood–brain barrier permeability, and the “Trojan horse” mechanism, whereby infected monocytes transport the virus across the BBB. Once in the CNS, WNV directly infects neurons, leading to apoptosis, excitotoxicity, and synaptic dysfunction. Meanwhile, astrocytes and microglia become activated and produce proinflammatory cytokines (e.g., TNF-α and IL-6) and chemokines, which amplify leukocyte recruitment but can also contribute to bystander neuronal injury (Potokar et al., 2019). Non-CNS cells, including dendritic cells and macrophages, influence the outcome by modulating systemic viremia and orchestrating innate and adaptive responses (Potokar et al., 2019; Stonedahl et al., 2020; Gern et al., 2024; Marshall et al., 2024). This dual action—direct viral cytotoxicity plus immune-mediated damage—underlies the severe neuropathological changes observed in WNV neuroinvasive disease.

In equines, symptoms include behavioral changes, ataxia, facial muscle twitching, dysphagia, and paralysis, sometimes progressing to coma or death (Angenvoort et al., 2013; Venter et al., 2017; Paré and Moore, 2018; Fehér et al., 2022). Birds display nonspecific symptoms as anorexia, ruffled feathers, dehydration with pathologies such as myocarditis, hepatitis, and retinal inflammation, especially in raptors (Gamino and Höfle, 2013). Neurological lesions and viral persistence in the CNS have been linked to lineage-specific pathogenesis (Williams et al., 2014). Infections with lineage 1 typically involve nephritis, hepatitis, and splenic lymphoid depletion, while lineage 2 infections are more often associated with myocardial necrosis and hepatic alterations (Williams et al., 2014).

### 7.2 Diagnosis of WNV

Definitive WNV diagnosis requires laboratory confirmation, typically via virus isolation or detection of neutralizing antibodies in serum or cerebrospinal fluid (CSF). Methods include hemagglutination inhibition, complement fixation test, and ELISA (Muerhoff et al., 2004; Sambri et al., 2013; CDC, 2024). Caution is required when interpreting diagnostic tests. For example, serological assays can show cross-reactivity with other flaviviruses, which can complicate the diagnosis. Viremia in humans is usually short-lived, which limits the window for direct virus detection. In immunocompromised individuals, including those receiving B-cell-depleting therapies, viremia can be prolonged. This necessitates repeated testing or reliance on neutralizing antibody detection. Additionally, the timing of sample collection relative to symptom onset significantly impacts sensitivity. CSF testing may be necessary in cases of suspected neuroinvasive disease (D’Arena et al., 2025). RT-PCR is the preferred detection method (Styer et al., 2007).

### 7.3 History of WNV global spread

First isolated in Uganda in 1937 (Sejvar, 2003; Kramer et al., 2007), WNV remained endemic in Africa and the Middle East until its emergence in America in 1999 (Hayes, 2001; Sejvar, 2003). Within a decade, it spread across the continent, with transmission also reported via transfusion, transplantation, and breastfeeding (Hayes et al., 2005; Kramer et al., 2007). WNV has now spread throughout most of the world, except Antarctica, and is considered one of the most widely distributed arboviruses and the leading cause of mosquito-borne viral encephalitis in humans. However, other etiologies causing encephalitis are more frequent globally, and their relative importance varies by region (Karim and Bai, 2023).

In Europe, the WNV first emerged in Romania in 1996, marking one of the earliest documented incursions into the continent (Savage et al., 1999). Since 2010, outbreaks have increased in several European countries, including Greece, Spain, and Italy. More recently, outbreaks have occurred in Germany and northern Europe, peaking in 2018 with 1,549 human cases and 166 deaths (Martín-Acebes and Saiz, 2012; Barrett, 2018; Ziegler et al., 2019). In 2023, over 700 cases were reported across Europe, with Italy accounting for over half of them (CDC, 2024). Spain first diagnosed WNV in 2004, though lineage 1 likely circulated since the 1960s. Endemic areas include the wetlands along the Guadalquivir River in Andalusia, which support abundant vector populations. Major outbreaks occurred in 2010 and 2020; the latter resulted in over 70 human cases and eight deaths, according to the Spanish Ministry of Health (D’Amore et al., 2022; Ministerio de Sanidad, 2025).

Ongoing climate change and migratory birds, such as blackbirds, magpies, and sparrows, are facilitating the continued spread of WNV across Europe (Bakonyi et al., 2006; Semenza et al., 2016; Mrzljak et al., 2020), similarly to how the American robin has been identified as a key reservoir in North America (Colpitts et al., 2012; Redazione, 2016; Semenza et al., 2016).

## 8 Prevention and control

**Figure 7:**
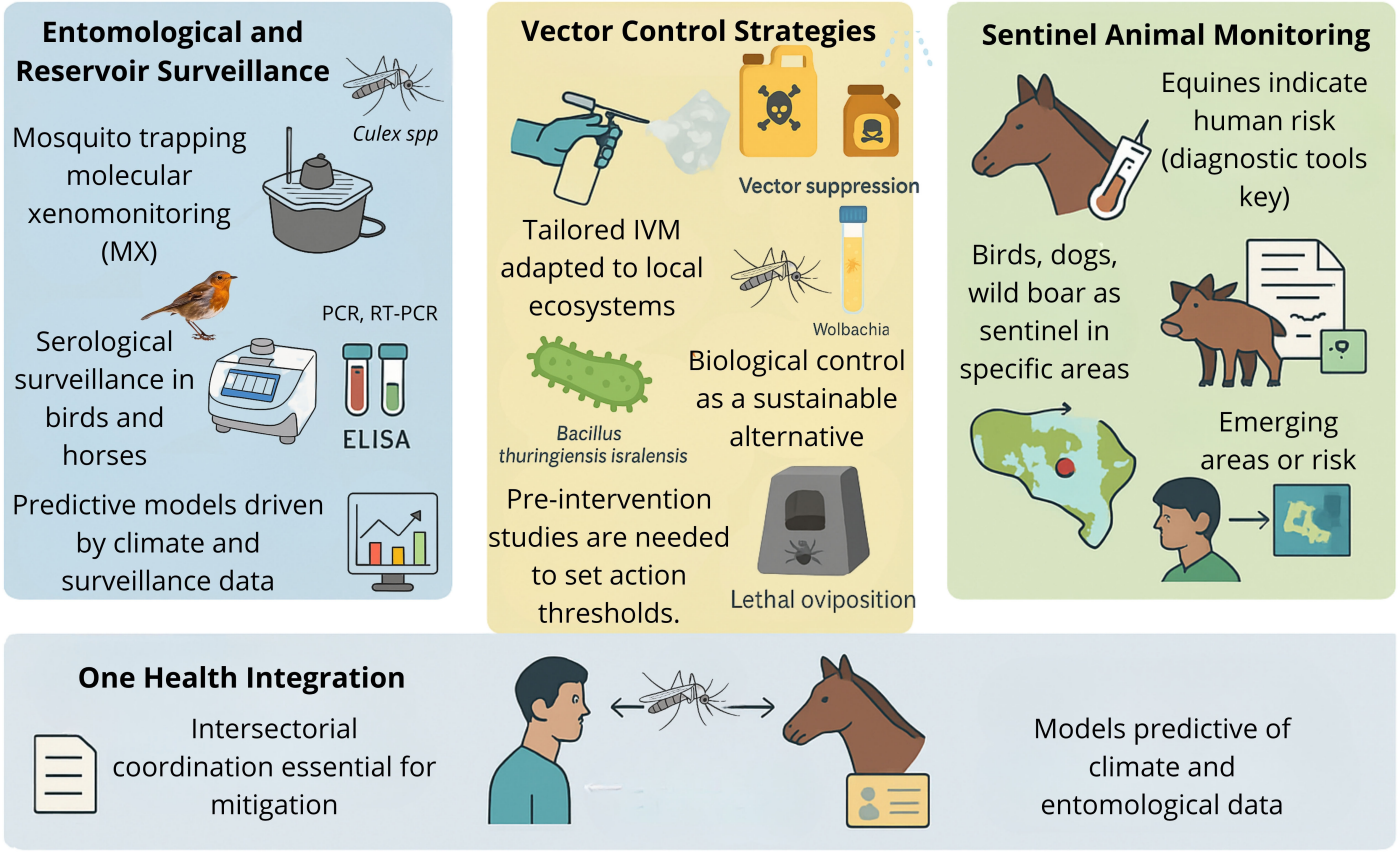
Prevention and control strategies within a One Health approach. Surveillance, control, and integration strategies for WNV prevention. Left panel: entomological and reservoir surveillance includes mosquito trapping, molecular xenomonitoring, and serological testing in birds and horses, supported by climate-driven predictive models. Center panel: vector control strategies involve tailored integrated vector management, biological control (e.g., *Bacillus thuringiensis israelensis*, Wolbachia), and lethal oviposition, with action thresholds established through pre-intervention studies. Right panel: sentinel animal monitoring uses equines, birds, dogs, and wild boar to detect emerging risk areas. Below: One Health integration promotes intersectoral coordination and predictive models combining climatic and entomological data.

Rising global temperatures, milder winters, and increased tropical rainfall have expanded the range of mosquito species, facilitating the emergence of vector-borne diseases. These currently account for nearly 30% of emerging infectious diseases (Reperant, 2010), with increasing global distribution and recurrent outbreaks (Gubler, 1996; Gould et al., 2017; Mayer et al., 2017). WNV is now the most widely distributed mosquito-borne virus (MBV) (Chancey et al., 2015). As no licensed human vaccine or antiviral treatment exists (De Filette et al., 2012; Ulbert, 2019), vector control remains the primary prevention strategy (Frasca et al., 2024). (**Figure 7**).

The prevention of WNV relies on integrated strategies that target vectors, reservoirs, and susceptible hosts. Vector control includes reducing larval habitats, controlling adult mosquitoes with insecticides, and taking personal protective measures, such as using repellents and bed nets. Combined mosquito monitoring (PCR, RT-PCR, and cell culture), reservoir serology (birds and horses), and climate analysis are essential components of surveillance programs for risk assessment and predictive modeling (Bakhiyi et al., 2025). However, despite these integrative approaches, WNV prediction models have shown limited success in accurately forecasting the timing and intensity of outbreaks, reflecting the complexity of ecological and climatic drivers (Holcomb et al., 2023b).

Vaccination is a key preventive measure. Several equine vaccines are licensed and effective. Human vaccine candidates, including DNA-, inactivated-, and mAb-based approaches, are under development or in early-phase clinical trials (Calvert et al., 2025; D’Arena et al., 2025). Pre-exposure prophylaxis with vaccines or monoclonal antibodies is being explored for high-risk populations. Post-exposure therapies, including the passive transfer of high-affinity neutralizing antibodies, have shown efficacy in experimental models.

Interdisciplinary “One Health” coordination is critical for early detection and outbreak mitigation (Bruno et al., 2025). This approach integrates human, animal, and environmental surveillance, enabling timely public health interventions. Additionally, stringent screening of blood products and organ transplants is essential to prevent iatrogenic transmission. Public education on personal protective measures, prompt recognition of clinical signs, and reporting of animal cases can further enhance control efforts.

### 8.1 Entomological and reservoir surveillance

Although WNV lacks pandemic potential, its 1999 emergence in New York initiated a widespread US epidemic, facilitated by Culex mosquitoes and over 300 avian hosts (Pierson and Diamond, 2020). The American robin (*Turdus migratorius*) played a central ecological role in viral amplification (Brüssow and Figuerola, 2025). This has been linked to a mutation in NS3 (T249P), which increases virulence in birds and a subsequent envelope gene mutation that enhances vector infectivity (Brault et al., 2007).

Recent data demonstrate the predictive value of both adult and immature mosquito surveillance for early outbreak detection, enabling timely interventions (Bakhiyi et al., 2025). Molecular xenomonitoring (MX), which analyzes mosquito excreta for viral RNA, provides a cost-effective and sensitive alternative (Bigeard et al., 2024). Integrating mosquito trapping with serological monitoring of sentinel birds enhances detection capacity (Ergunay et al., 2014).

Integrated surveillance annual programs (May–September) should combine mosquito monitoring (PCR, RT-PCR, cell culture), reservoir serology (birds, horses), and climate analysis to construct predictive models (Bakhiyi et al., 2025). Nevertheless, models for predicting WNV outbreaks have shown limited effectiveness in accurately forecasting the timing and magnitude of outbreaks when integrative approaches are applied. This underscores the inherent complexity of ecological and climatic determinants (Holcomb et al., 2023a).

Effective early detection and risk mitigation require coordinated, interdisciplinary efforts aligned with the One Health framework. Interdisciplinary coordination (One Health) is essential for early detection and risk mitigation. Implementing integrated surveillance requires developing structured models that go beyond conceptual frameworks. Recent research by Bruno et al. (2025) presents a One Health/Eco-Health approach in which veterinary, human, and environmental health data are systematically integrated into a single platform to enable the early detection of risks and interventions (Bruno et al., 2025). The model prioritizes harmonizing diagnostic systems, establishing shared data infrastructures, and actively involving communities. This demonstrates that coordinated surveillance improves the prediction and management of outbreaks. These operational models offer a practical foundation for improving preparedness for the West Nile virus in endemic and newly affected regions.

### 8.2 Vector control: strategies and effectiveness

Vector suppression via aerial spraying, larvicides, and adulticides has proven effective in the US southern states (Nasci and Mutebi, 2019). Vector control interventions using aerial spraying, larvicides, and adulticides have been shown to be effective in the southern US (Hayes, 2001). However, the aerial and truck-based application of adulticides often raises concerns about environmental and human health safety. Importantly, existing evidence reveals no consistent link to adverse health outcomes when these measures are conducted in line with established guidelines. This indicates that the perceived risks often outweigh those documented by current research (Mendoza et al., 2023). In addition, larval control programs conducted in the state of Connecticut, reduced Culex densities and altered its entomological presence (McMillan et al., 2021). However, resistance development (Atyame et al., 2019) and environmental conditions such as temperature (Kalmouni et al., 2024) must be considered. Tailored Integrated Vector Management (IVM) programs, adapted to local ecosystems, are recommended (Bellini et al., 2014).

Biological control strategies, including *Bacillus thuringiensis israelensis*, have shown success (Hongoh et al., 2016). Additional measures involve aquatic and aerial predators (Canyon and Hii, 1997), source reduction, lethal oviposition traps, and novel methods like Wolbachia-infected mosquito release, which impairs viral replication (Jeffries and Walker, 2016; Yen and Failloux, 2020; Zhang et al., 2020).

In summary, effective implementation of vector control strategies requires pre-intervention studies and ecologically sound control thresholds based on entomological data, infection rates, and climate. Despite the promising results available, few studies have quantified reductions in human cases, highlighting the need for continuous, multidisciplinary, and well-funded surveillance systems.

### 8.3 Role of sentinel animals

WNV emergence in new regions demands early detection strategies. Molecular diagnostics are preferred over serology due to cross-reactivity among orthoflaviviruses (Zeller, 1999). Since 2000, the US CDC has used sentinel birds and equines for early warning (Centers for Disease Control and Prevention (CDC), 2000; Centers for Disease Control and Prevention (CDC), 2001; Centers for Disease Control and Prevention (CDC), 2002; CDC, 2024). Equine and human infections share clinical and immunological features (Schwarz and Long, 2023), allowing equine surveillance to inform human risk (Yuen et al., 2024). Seroprevalence studies in Europe support equine monitoring as a predictive tool (Gothe et al., 2023; Rusenova et al., 2024). ELISA-based IgG/IgM detection demonstrated high accuracy. Other sentinel candidates, such as dogs and wild boars, have also proven effective in peri-urban environments (Holicki et al., 2025).

## 9 Current and experimental anti-WNV strategies

### 9.1 Peptides and small molecules as therapeutic agents

Antiviral strategies against orthoflaviviruses target key replication cycle stages via S proteins interacting with membranes or NS proteins that inhibit replication and/or cell entry (Munjal et al., 2017). Antiviral peptides exhibit high specificity and low toxicity, demonstrating efficacy in preclinical models, but their clinical potential is limited by rapid degradation, short half-life, and poor bioavailability (Bai et al., 2007; Shi, 2009). Structural modifications, including cyclization, nanocarrier conjugation, nanoparticle encapsulation, and biodegradable polymers, improve stability, half-life, and delivery (Mertinková et al., 2021; Xiao et al., 2025).

Phage display libraries identified WNV-targeting peptides, such as P1 (IC_50_ = 67 μM) and its derivative P9 (IC_50_ = 2.6 μM), which conferred complete protection in murine models and crossed the BBB (Bai et al., 2007). DIII and DII hinge regions of the E glycoprotein remain promising targets, with computational and combinatorial approaches generating peptides that inhibit neuroinvasion (Nybakken et al., 2006; Bai et al., 2007; Costin et al., 2010; Mertinková et al., 2021; Saiz et al., 2021).

Small-molecule inhibitors targeting NS2B–NS3 protease, NS3-NS5 interactions, and NS5 polymerase have shown efficacy *in vitro* and *in vivo*, including compounds like C-30 (ZINC19598270), which reduced viral loads in spleen and brain (Fernandes et al., 2021; Celegato et al., 2023; García-Zarandieta et al., 2023; Kocabiyik et al., 2025). High-throughput screening of drug libraries focuses on viral enzymes, entry, and maturation pathways (García-Serradilla et al., 2019; Li and Peng, 2021; Namasivayam et al., 2022; Grabski et al., 2024; Zheng et al., 2024; Lissane Eddine et al., 2025).

As of 2025, no peptide or small-molecule therapy has been clinically approved for WNV, though preclinical results underscore their therapeutic promise, with challenges remaining in stability, delivery, and brain penetration (Zheng et al., 2024; Kocabiyik et al., 2025).

### 9.2 RNA structural elements as potential antiviral targets.

WNV fitness depends on conserved RNA structural elements, making them attractive targets for antiviral strategies that disrupt RNA structure or interactions. Nucleic acid–based approaches—including antisense oligonucleotides, ribozymes, aptamers, and siRNAs—have demonstrated preclinical efficacy and early-phase clinical potential.

Antisense locked nucleic acids (ASO-LNAs) have been used to disrupt specific RNA elements, showing therapeutic promise across the WNV genome (Huston et al., 2024). Antisense phosphorodiamidate morpholino oligomers (PMOs) targeting the 5’ end or 3’ UTR efficiently reduced viral titers *in vitro*, and conjugation with arginine-rich peptides enhanced uptake, partially protecting mice (Deas et al., 2005; Deas et al., 2007). Similarly, siRNAs targeting viral RNA achieved >90% inhibition in culture and significant protection in murine models (McCown et al., 2003; Bai et al., 2005; Geiss et al., 2005; Kumar et al., 2006; Ye et al., 2011; Karothia et al., 2025).

Chemically modified aptamers, selected for high-affinity binding to viral RNA, increased survival of WNV-infected mice by up to 80%, demonstrating partial protection against lethal infection (Yang et al., 2006; Marton et al., 2010). These studies highlight RNA-targeting molecules as promising antiviral candidates against WNV.

### 9.3 Other Strategies against West Nile fever

Monoclonal antibodies (MAbs) represent a promising therapy for the prevention and treatment of neuroinvasive neurotropic encephalitis (NNE) caused by viruses in immuno-compromised individuals, although penetration across the BBB remains a major limitation (Calvert et al., 2025). Type I IFN plays a critical role in antiviral immunity (Weber et al., 2014). Mice or humans with compromised type I IFN responses are highly susceptible to severe WNV disease (Daffis et al., 2009; Gervais et al., 2023; Lin et al., 2023). Prophylactic (pre-infection) administration of type I IFN in cell culture and animal models confers protection against WNV, with IFN-β showing the strongest antiviral effect *in vitro* (Daffis et al., 2009; Ferrari et al., 2025).

Metabolic studies have revealed virus-induced glycolytic shifts, suggesting novel therapeutic targets (Mingo-Casas et al., 2023). Currently, treatment is limited to supportive care and experimental use of IVIG and IFN-α (Mbonde et al., 2024). Interferon-α-2b has shown inhibition of WNV replication *in vitro*, and limited clinical reports suggest possible benefit if administered early after symptom onset, particularly for WNV neuroinvasive disease. However, its effectiveness may be reduced once viral replication is established due to viral strategies that block IFN signaling (Daffis et al., 2009; Shi, 2009; Ferrari et al., 2025). To date, no vaccines or antiviral agents have advanced beyond Phase II clinical trials (Ronca et al., 2021). Further clinical development of promising candidates is essential (see section 9.3).

### 9.4 Lethal mutagenesis

Lethal mutagenesis offers an alternative to classical antiviral inhibitors by exploiting RNA viruses’ high mutation rates. Mutagenic compounds induce excessive genomic errors, reducing infectivity and potentially driving virus extinction, a phenomenon termed error catastrophe (Perales and Domingo, 2016; Swanstrom and SChinazi, 2022). This strategy limits resistance emergence, as surviving viral genomes remain impaired, and highly mutagenized genomes can interfere with replication of less-mutated viruses, known as lethal defection (Arias et al., 2014; Díaz-Martínez et al., 2018).

Favipiravir and molnupiravir are promising mutagens for WNV. Favipiravir, a pyrimidine analogue, increases viral mutations *in vitro* and has been effective against related neurovirulent orthoflaviviruses (Escribano-Romero et al., 2017; Bologheanu et al., 2020). Molnupiravir crosses the blood–brain barrier and shows efficacy against neuroinvasive viruses, although not yet tested in WNV (Swanstrom and SChinazi, 2022; Ojha et al., 2024). Ribavirin has shown mixed outcomes against WNV, highlighting the need for further evaluation of mutagenic compounds alone or in combination (Alli et al., 2021; Hadj Hassine et al., 2022).

## 10 Current challenges and future perspectives

Despite extensive research on WNV, no specific antiviral therapies or licensed human vaccines are currently available, leaving vector control and personal protective measures as the primary means of prevention. An overview of current knowledge gaps and proposed research directions to address future challenges in the control, treatment, and understanding of WNV is summarized in **Table 1**.

**Table 1:** Key knowledge gaps, proposed research directions, and priority areas for advancing West Nile virus (WNV) research.

Knowledge gap/challenge	Proposed research direction	Priority area
Incomplete understanding of WNV quasispecies and intra-host diversity dynamics	Genomic studies using NGS to identify minority variants and track evolution in hosts and tissues	Virology/Evolution
Limited data on RNA structural elements and their role in viral replication and host adaptation	Functional characterization of UTRs and coding RNA elements to guide antiviral design	Molecular biology/Therapeutics
Lack of a human vaccine	Development of vaccines based on structural epitopes (E protein DIII) and NS1-based platforms	Vaccine development
Insufficient understanding of immune evasion and post-translational modifications	Characterization of ubiquitination, SUMOylation, and phosphorylation in immune modulation to identify targets	Immunology/Host–pathogen interaction
Lack of broad-spectrum antivirals targeting essential non-structural proteins	Design of inhibitors targeting NS3/NS5 enzymatic activities or NS2B–NS3 interaction; evaluation of lethal mutagenesis	Antiviral therapy
Impact of climate change on WNV epidemiology is poorly quantified	Predictive models integrating climate, vector ecology, and transmission data	Ecology/Epidemiology
Lack of integrated One Health surveillance systems	Real-time networks combining entomological, avian, veterinary, and human health data	Public health/One Health
Underexplored structural dynamics of viral proteins in infection and antibody evasion	Cryo-EM and structural studies to guide epitope mapping and antibody development	Structural Biology/Immunology

The table outlines current challenges in understanding WNV biology, immunity, and epidemiology, alongside strategic directions aimed at improving prevention, therapeutic development, and surveillance under a One Health framework.

Nevertheless, the lack of standardized tools for rapid WNV diagnosis constitutes a major limitation to effectively apply antiviral therapies, and thus the development of rapid point-of-care diagnostics is critical to control infection and disease (Lustig et al., 2018; Mbonde et al., 2024). Novel approaches based on real-time reverse-transcription loop-mediated isothermal amplification or on CRISPR-Cas12a methods are offering promise in the development of early detection tools for WNV infection ((PDF) A novel CRISPR-Cas12a-based diagnostic for rapid and highly sensitive detection of West Nile Virus; Khedhiri et al., 2024).

### 10.1 Research needs in viral genetics and quasispecies

Despite extensive WNV research, key gaps remain in understanding quasispecies dynamics and evolution. Comprehensive profiling of viral populations in both vertebrate and invertebrate hosts is needed, as host-dependent bottlenecks and selection pressures influence viral diversity (Brackney et al., 2011; Dridi et al., 2015; Caldwell et al., 2020). Ultra-deep sequencing and standardized bioinformatic pipelines could clarify minor variants’ roles in neuroinvasiveness, immune evasion, and vector competence (Domingo and Perales, 2019; Saiz et al., 2021; Fitzmeyer et al., 2023).

Integrating genetic data with ecological and epidemiological models, including climate, host migration, and vector control, can improve outbreak prediction (Ruiz-López et al., 2023). Understanding host immune responses, especially type I interferons, OAS/RNase L, and antibody pathways, is critical for vaccine and therapeutic development (Ahlers and Goodman, 2018; Habarugira et al., 2020; Kocabiyik et al., 2025). Global genomic surveillance is essential to detect emerging escape variants. Interdisciplinary approaches combining molecular virology, computational biology, vector ecology, and public health are crucial to modeling WNV evolution and mitigate disease impact.

### 10.2 Preparedness for future outbreaks within One Health approach

Effective preparedness for WNV requires integrated surveillance, community engagement, and targeted interventions. Advanced systems combining real-time mosquito monitoring, human case reporting, and AI-driven analytics enhance early detection, hotspot mapping, and resource allocation (Gupta et al., 2025). Integration of entomological and epidemiological data allows prediction of outbreaks and informs vector control strategies, accounting for environmental factors such as temperature and precipitation.

Challenges persist, including underreporting, reporting delays, and variability across jurisdictions (CDC, 2025). Sustained funding, scalable technologies, and public engagement are essential. Preparedness also relies on integrated pest management strategies, including source reduction, larvicide, and targeted adult mosquito control, combined with ongoing research and adaptive planning to address climate-driven shifts in transmission risk (Hongoh et al., 2016).

### 10.3 Perspectives on human vaccine development

Effective WNV vaccine development requires detailed knowledge of viral biology and immune mechanisms, including identification of protective antigens and mechanistic correlates of immunity (Britto and Alter, 2022; Kumar et al., 2025). Despite vaccines for other orthoflaviviruses (DENV, YFV, JEV), none are approved for WNV (Bello et al., 2024). Several candidates—including live attenuated chimeric, DNA, and subunit vaccines—have shown immunogenicity in early trials, with ChimeriVax-WN02 demonstrating high seroconversion and favorable safety (Kocabiyik et al., 2025). DNA vaccines targeting prM and E proteins provided full protection in animal models (Davis et al., 2021).

Challenges include ensuring safety in older adults, low and fluctuating disease incidence, and limited commercial interest (Kocabiyik et al., 2025). Progress will rely on innovative trial designs, advanced vaccine platforms, and collaborative efforts to achieve a safe, effective, and cost-efficient WNV vaccine, especially amid climate-driven transmission risks (Kocabiyik et al., 2025).

## 11 Conclusions

WNV exemplifies an adaptable and pathogenic emerging arbovirus, with ecological flexibility, human and animal virulence, and complex host immune interactions posing ongoing global health challenges. This review examines WNV at molecular, genetic, and epidemiological levels, emphasizing how intra-host diversity and quasispecies dynamics influence evolution, virulence, and immune evasion, critical for vaccine and therapeutic design.

WNV replicates across diverse hosts, from mosquitoes to vertebrates, maintaining pathogenic potential. Its adaptability derives from the error-prone NS5 RNA-dependent RNA polymerase, generating genetic variants responsive to selective pressures. However, variability within mosquito populations remains underexplored, representing a knowledge gap relevant to outbreak dynamics, transmissibility shifts, and neuroinvasive strain emergence, particularly under climate-driven vector expansion.

Immunologically, WNV persistence reflects both replication efficiency and modulation of innate responses. Viral proteins, including NS1 and NS5, affect IFN signaling and complement pathways, while structured RNA elements and post-translational modifications enhance immune evasion, identifying potential antiviral targets.

Despite widespread distribution and increasing impact, no human vaccine exists, and treatment remains supportive. The recent identification of WNV lineage 3 in a human case in Nebraska underscores that less common lineages should not be dismissed as incidental findings. This event highlights the importance of molecular surveillance to detect emerging variants with potential clinical relevance.

Addressing WNV necessitates integrated strategies: vaccine development targeting conserved epitopes, antivirals exploiting replication fidelity limits, and robust One Health–based surveillance. Interdisciplinary approaches leveraging NGS, systems immunology, and bioinformatics can track viral diversity, predict evolutionary trends, and guide precision interventions. WNV underscores the need for coordinated proactive responses to emerging pathogens amid ecological and climate uncertainties.
